# Stratification of the Extent of Visual Impairment Identifies Sex-Specific Degenerative Changes in Retinal Structure and Function during Aging

**DOI:** 10.31083/JIN25805

**Published:** 2025-03-04

**Authors:** Genea Edwards, Sean M. Riordan, Caitlin Buchholz, Marc Mardelli, Carlyn P. Euritt, Rodrigo Perez-Magnelli, Ariej Rafiq, Avery Engelmeyer, Peter Koulen

**Affiliations:** 1 Department of Ophthalmology, Vision Research Center, School of Medicine, University of Missouri-Kansas City, Kansas City, MO 64108, USA; 2 Department of Biomedical Sciences, School of Medicine, University of Missouri-Kansas City, Kansas City, MO 64108, USA

**Keywords:** C57BL/6J, aging, retina, vision, optomotor reflex, spectral-domain optical coherence tomography, electroretinogram

## Abstract

**Background:**

Initial manifestations of neurodegenerative ocular conditions, including age-related macular degeneration (AMD) and glaucoma, often remain undetected in the early stages and can begin after the age of 50 years with the likelihood gradually increasing each year thereafter. This study aimed to explore variances in visual and retinal function and anatomy among C57BL/6J mice, aiming to pinpoint differences between biological age and sex factors that potentially lead to the onset of vision impairment.

**Methods:**

A longitudinal study evaluated visual acuity (VA) and contrast sensitivity (CS) using optomotor reflex (OMR), and retinal function, encompassing scotopic and photopic measurements, was recorded by electroretinogram (ERG) at 12 months of age. Tissue was subsequently harvested for histological analysis, complementing the *in vivo* findings. Disparities in visual function were observed between individual male and female mice, necessitating categorization of visual impairment levels to investigate further sex-specific differences in the study’s aging population. Comparisons between sex and the degree of visual impairment were conducted using ANOVA followed by Tukey’s or Bonferroni’s post-hoc corrections and unpaired *t*-tests. Pearson correlation analysis determined the association between biological factors.

**Results:**

Sex-related disparities were found in the visual function of male (n = 13) and female (n = 18) mice aged 5–12 months. Eyes were categorized by vision impairment: normal vision, or low, moderate, or severe vision loss at the end of the study. Male and female mice differed in mean contrast sensitivity, indicating less sensitivity to fine detail and moving stimuli in female mice (11–12 months old, *p* < 0.001). Spectral-domain optical coherence tomography (SD-OCT) revealed a thinner retinal outer nuclear layer in male mice (*p* < 0.0001), although this did not vary across different levels of vision impairment. ERG indicated slower retinal responses in male mice (*p* < 0.05), while histology showed a significant reduction in the inner plexiform layer thickness in male mice with severe vision loss (*p* < 0.0001). Conversely, female mice exhibited greater thinning in the photoreceptor layer when vision was unimpaired (*p* < 0.01).

**Conclusions:**

The study shows that sex and extent of vision impairment influence visual and retinal health, with individual retinal layers differentially changing in thickness over time.

## 1. Introduction

The inbred C57BL/6J wild-type mouse is a staple in research, used in various research areas, including neurobiology, diabetes, obesity, cardiovascular, developmental, immunology, and genetics [1–7]. Comparative studies encompassing behavior, aging, and or sex differences are necessary to understand the cause or severity of common diseases and treatment outcomes [8–11]. Mice are advantageous models for aging studies because of their relatively short lifespans compared to humans. Despite the widespread use of the C57BL/6J mouse model in preclinical research and the recognition of age and sex as critical biological variables in humans, researchers have yet to determine the impact of age and sex on many physiological and pathophysiological processes studied with this model.

Age represents a biological variable that affects a similarly wide range of physiological and pathophysiological processes and encompasses both the development and aging of the structure and function of organs. Aging is a significant contributor to the accumulation of oxidative stress that results in degeneration of the structure and function of tissues, including the retina [12]. Oxidative stress-induced cellular senescence and reduced density of retinal ganglion cells, bipolar cells, photoreceptors, and pigment epithelial cells can induce vision loss [13,14]. Structurally, retinal layer thickness in normal eyes changes with age, specifically significant decreases in the thickness of the peripapillary retinal nerve fiber layer (RNFL), pericentral ganglion cell layer (GCL), peripheral inner plexiform layer (IPL), and foveal outer segment layer (OSL) thickness with age, and a significant increase in the thickness of the foveal retinal pigment epithelial (RPE) with age [15]. Similarly, Won and colleagues determined age-related changes in the thickness and volume of retinal layers, specifically thinner peripheral RNFL, GCL, and pericentral and peripheral IPL and thicker foveal inner nuclear layer (INL) and inner retina (IR) in the older group (>60 years) when compared to the younger group (<30 years) [16]. Early degenerative changes can vary in onset age depending on the specific condition. Typically, these changes begin to manifest in adults aged fifty and older. However, most individuals do not notice symptoms until age 55 or older [17].

Sexual dimorphism appears in many biological processes, including visual function, where factors such as disease susceptibility, color perception, visual acuity (VA), and photoreceptor cell distribution and density vary depending on sex [18]. Male humans possess a thicker macula [19], a more significant relative number of long wavelength sensitive (L-cones) and middle wavelength sensitive (M-cones) cone photoreceptors [20], increased response to blue light stimulation [21], and significantly heightened retinal sensitivity for fine detail and fast-moving stimuli [22,23]. Increased iron levels are found in the female retina and RPE [24,25]. The most well-known dimorphism is color perception. X-linked red-green colorblindness found in males [26] is the most common, while females are more prone to age-related macular degeneration (AMD) [27,28] and glaucoma [29]. Metabolic profiles from different ocular tissues display sexual dimorphisms [18]. Sex-related structure and function differences in the retina are likely due to hormone profiles [30]. Study has shown that female mice exhibit larger amplitudes in electroretinogram (ERG) recordings than male mice [31]. Spectral-domain optical coherence tomography (SD-OCT) has shown differences in the mean thickness of the outer nuclear layer (ONL), the outer plexiform layer (OPL), and the INL in the macular region, while the RNFL is thicker in females [30]. The estrus cycle may exert a role in these structural and functional differences due to the presence of estrogen receptors in ocular tissues [32]. The menstrual cycle and accompanying hormonal fluctuations modulate ocular structures, including the retina [33]. However, the exact mechanism and implications of these changes are still a topic of ongoing research.

In addition to the significant impact of both age and sex as biological variables, one of the challenges to the development of new therapeutics for age-related neurodegenerative diseases of the retina, such as dry AMD and glaucoma, is the lack of animal models that recapitulate underlying mechanisms of, and phenotypes present in retinal disease. In many studies, animals are genetically modified or exposed to risk factors such as advanced aging or specific diets to establish the pathobiological processes involved with the expectation of treating or delaying its progression [34–38]. Rigorous studies of wild-type mice are needed to understand better time-related changes that potentially predispose to or contribute to neurodegenerative pathology. Visual processing of moving stimuli evaluated by initial and late phase optokinetic responses deteriorates in aged (21–24-month-old) mice compared to young adult mice [39]. A study utilizing C57BL/6J mice aged between 2 and 32 months reported that natural age-related retinal function and morphology changes occur gradually rather than abruptly [40]. Additionally, other studies described morphological changes, including inappropriate localization of photoreceptor synapses [39], topographic differences in RPE morphology [41], neuroinflammation, and increased glial reactivity in C57BL/6J mice [42]. As new models of retinal degeneration are developed, recent advancements in retinal organoid cultures have emerged as a particularly exciting area of research. These three-dimensional tissue constructs hold significant potential for applications in personalized medicine, drug screening, gene therapy, and cell transplantation. Retinal organoids provide a more precise model for studying human retinal diseases, serving as a valuable complement to traditional animal studies, particularly when animal models exhibit only partial traits of specific disease phenotypes [43,44].

Our study evaluated visual performance longitudinally in wild-type, middle-aged C57BL/6J mice aged 5–12 months old, retina structure and function using SD-OCT imaging and ERG recordings at the completion of the study, with post-mortem histological analysis. This work sought to clarify early age-related changes in visual and retinal function and structure in the retina of wild-type mice, routinely used in retinal neurodegenerative research and as a potential model for assessing age and sex as biological variables. Empirical stratification of visual impairment, as an additional variable, provides a framework for understanding visual impairment in the early stages of the neurodegenerative process by evaluating sex-dependent differences in visual impairment results.

## 2. Materials and Methods

### 2.1 Animals

Five-week-old C57BL/6J male (n = 13) and female (n = 18) mice were purchased from Jackson Laboratories (Bar Harbor, ME, USA). All animals were socially housed in plastic shoebox cages at 25 °C, with ad libitum access to Teklad Global 18% Protein Rodent Diet (Envigo, Somerset, NJ, USA; Cat. #2918.15) and water and maintained on a 12-hour light/dark cycle. Weekly body weights of all animals were recorded, and the average weights were plotted in graphs at two-month intervals. The mice underwent visual performance screenings at 5 months old until they reached 12 months old. Each animal’s right and left eyes were tested separately, depending on the direction of the stimulus pattern, with each eye representing n = 1. Each eye was considered individually for statistical analysis and to capture individual differences in the results. Eyes were removed from the study if found to have ocular injuries, opacities, or abnormal ocular anatomy to ensure accurate and reliable results [45–47]. A total of 48 eyes were included after excluding ineligible samples.

Behavioral VA assessments using the optomotor response aided in categorizing individual mouse eyes into 4 groups: normal vision (>0.361 c/d; n = 7), low vision loss (0.301–0.360 c/d; n = 18), moderate vision loss (0.240–0.300; n = 15), and severe vision loss (0.000–0.239; n = 8). The spatial frequency scale, or VA, is generally based on visual thresholds determined in C57BL/6 mice by Prusky and colleagues, where the threshold reached 0.400 c/d by P25 and remained constant into adulthood (P90-P125) [48]. The Institutional Animal Care and Use Committee (IACUC) at the University of Missouri-Kansas City (protocol 1902-02) approved all experimental animal procedures following institutional and federal guidelines and the Association for Research in Vision and Ophthalmology (ARVO) Statement for the Use of Animals in Ophthalmic and Vision Research. At the predetermined endpoint, euthanasia of study animals was conducted using 30% chamber vol/min CO_2_ inhalation, followed by cervical dislocation as a secondary method to confirm death.

### 2.2 Behavioral Assessment of Visual Function

Visual acuity or spatial frequency and contrast sensitivity (CS) assessment were determined using the OptoMotry© optomotor testing system with OptoMotry software, version 1.7.7 (CerebralMechanics Inc, Lethbridge, Alberta, Canada) described earlier [49–52]. Briefly, testing was conducted monthly between 5 and 12 months of age. Each measurement was averaged and plotted in graphs at two-month intervals. Visual acuity is the maximum spatial frequency in which an optomotor response and head tracking has occurred. Visual acuity was measured at 100% contrast, using a drift speed of 12.0 d/s and a cutoff maximum of 0.500 c/d. Contrast sensitivity refers to the minimum contrast level at which head tracking is detected. A lower contrast percentage indicates better contrast vision. The measurement of contrast sensitivity began at a baseline spatial frequency of 0.042 c/d (cycles/degree) and a beginning contrast level of 100%. Animals were placed on a 55 mm or larger platform (dependent on weight) where stimuli of varying spatial frequencies or contrast levels elicited a response [48,53].

### 2.3 Electroretinography

Electroretinography responses were recorded with an HMsERG electroretinography system with accompanying software, version 4.180 (Ocuscience, Henderson, NV, USA), as described previously [51,52]. The retinal function of rods (scotopic) and photoreceptors (photopic) was determined between 12–15 months of age. Animals were dark-adapted overnight, with only red light used for setup and performing the test. Anesthesia was initially administered at 4.0% isoflurane (cat. #029405, Covetrus, Dublin, OH, USA) with an oxygen flow rate of 1 L/min and maintained at 2.0% with the same oxygen flow rate for the test duration. Anesthetic drops of 0.5% Proparacaine Hy drochloride Ophthalmologic solution (Henry Schein, Inc., Cat. #2963726, Port Washington, NY, USA), dilation drops of 1% Tropicamide solution (Henry Schein, Inc., Cat. #70069012101, Port Washington, NY, USA), and 0.3% hypromellose (GenTeal^®^ Tears Ophthalmic Gel, Henry Schein, Inc., Cat. #0065806401, Port Washington, NY, USA) eye drops were added to each eye for corneal lubrication. A warming pad maintained the body temperature at 37 °C while a temperature probe monitored the animal’s temperature. The ground electrode (Ocuscience, Henderson, NV, USA) was placed above the base of the tail, and reference electrodes (Ocuscience, Henderson, NV, USA) were placed behind the ear with the tip just behind each eye. Thread electrodes (Ocuscience, Henderson, NV, USA) and a 2.0 mm mini contact lens made of Aclar material were coated with GenTeal tears and placed on each eye to prevent moisture loss throughout the procedure.

ERG scotopic and photopic responses were acquired and analyzed using ERGView 4.380V software (Ocuscience, Henderson, NV, USA) accompanied by the HMsERG system using a 150 Hz low pass filter and 60 Hz noise-eliminating filter. The ERGView software determined the b/a wave ratio, which serves as an indicator of inner-to-outer retinal function. Scotopic threshold responses (STRs) and photopic negative responses (PhNRs) were calculated from flashes generated from the scotopic flash intensity series (−4.5 to −3.5 log cd*·*s/m^2^) and photopic flash intensity series (0.0 to 1.5 log cd*·*s/m^2^) as described previously [52].

### 2.4 Optical Coherence Tomography

*In vivo* imaging determined retinal thickness in wild-type C57BL/6J mice at 15 months. Optical coherence tomography images were obtained by an iVivo^®^ SD-OCT with OctEngine software, version 1.8.41.1 (Ocuscience, Henderson, NV, USA) with 5μm transverse resolution, submicron depth resolution, and a live fundus image-guided alignment. Pupils were anesthetized with 0.5% Proparacaine Hydrochloride Ophthalmologic solution (Henry Schein, Inc., Cat. #2963726, Port Washington, NY, USA) and dilated with 1% Tropicamide solution (Henry Schein, Inc., Cat. #70069012101, Port Washington, NY, USA). GenTeal^®^ Tears Ophthalmic Gel (Henry Schein, Inc., Cat. #0065806401, Port Washington, NY, USA) lubricated the cornea throughout the scans. A platform allowed researchers to manipulate the mice into the correct position for imaging. A SomnoFlow (Kent Scientific, Torrington, CT, USA) anesthesia system administered isoflurane (cat. #029405, Covetrus, Dublin, OH, USA) anesthesia and maintained it at 2.0% for the time needed to acquire images. The platform allowed anesthesia to flow through a nose cone for the imaging duration. Images were exported as JPEG and TIFF files to quantify the thickness of the retinal layers at 500 μm from the optic nerve. An average of 16 B-scan images were post-processed using VQ Enhance software, version 1, provided by Lumedica, Durham, NC, USA. Open-source Fiji Image J, 64-bit software (https://imagej.net/software/fiji/, NIH, Bethesda, MD, USA) was used to measure retinal thickness [54]. Total retinal thickness is defined as the distance from the RNFL (including the RNFL) to the RPE layer (including the RPE layer) in a vertical retina section. Transformation of greyscale images to pseudo-color was accomplished by applying a royal lookup table (LUT) provided by VQ Enhance software.

### 2.5 Tissue Fixation and Staining

After euthanasia and assertion of death, eyes were enucleated and immersion-fixed in a 4% formaldehyde solution (16% w/v paraformaldehyde (PFA) aqueous solution, Electron Microscopy Sciences, Cat. #15710, Hatfield, PA, USA) in phosphate buffer (0.1 M PB, pH 7.4) for 30 min at 4 °C. After 30 min, a cut was made along the ora serrata, and the eyes were returned to the 4% formaldehyde solution for O/N fixation. The next day, the tissue was removed from the fixative and cryoprotected with increasing concentrations of 10% and 20% sucrose solutions in 1× phosphate buffered saline (PBS) at 4 °C until the tissue sank, lastly at 30% sucrose O/N and stored at 4 °C before embedding. The cornea, iris, and lens were dissected away, leaving only the eyecup to be embedded. Cryoprotected eyecups were embedded in an optimal cutting temperature medium (OCT, Fisher Scientific, Cat. #23-730-57, Pittsburg, PA, USA) and stored at −80 °C for 1–2 days. Vertical sections were cut into 10–14 μm sections via a Leica CM3050 S Cryostat (Leica Biosystems, Nusslock, Germany). A 4% formaldehyde solution was added to the tissue section for 15 min at RT before hematoxylin and eosin staining (H&E). OCT-embedded tissue sections were washed with 1× PBS, then hydrated with a 5 min wash in ddH_2_O. Hematoxylin staining was performed on retinal tissue sections for 30 s, rinsed with ddH_2_O, and then Scott’s Tap Water for 1 min. After washing with Scott’s Tap Water (cat. # 26070–06, Electron Microscopy Sciences, Hatfield, PA, USA), sections were stained with eosin for 30 s to assess retinal morphology. Tissue was then dehydrated in 95% ethanol before absolute ethanol and mounted with VectaMount Permanent Mounting Medium (Vector Laboratories, Inc., Cat. # H-5501–60, Burlingame, CA, USA).

### 2.6 Microscopy

A Leica DM IL LED inverted microscope (Leica Microsystems, Mannheim, Germany) and a Q imaging 12-bit camera (Q Imaging, Surrey, BC, Canada) acquired the light microscopy images at 4× and 20× magnification using QCapture software, version 2.9.13 (Teledyne Technologies, Surrey, BC, Canada). All images were saved as TIFF files for analysis. Three to six biological replicates were images in addition to three measurements per field, all within 500 μm of the optic nerve head.

### 2.7 Image Analysis

Open-source Fiji ImageJ Windows 64-bit software version (https://imagej.net/software/fiji/downloads) was used for image analysis, histological quantification of layer thickness, and photoreceptor nuclei counts. A representative image of a histological section from a 15-month-old female C57BL/6J mouse with severe vision loss (Supplementary Fig. 6A) demonstrates the quantification of retinal layers and the counting of photoreceptor nuclei rows (Supplementary Fig. 6B). Each image was quantified in triplicate.

### 2.8 Statistical Analysis

Differences between sexes and visual impairment from age-related changes were determined by a two-tailed, unpaired *t*-test or one- or two-way ANOVA with Tukey’s or Bonferroni’s post-test, where indicated using Graph Pad Prism^®^ 10.2.3 software (GraphPad, San Diego, CA, USA). Results were considered significant with a *p*-value of *p* < 0.05 (*) and highly significant if the *p*-value was *p* < 0.01 (**), *p* < 0.001 (***), *p* < 0.0001 (****).

## 3. Results

### 3.1 Differences in Body Weight for Middle-Aged C57BL/6J Male and Female Mice

**Fig. 1.**
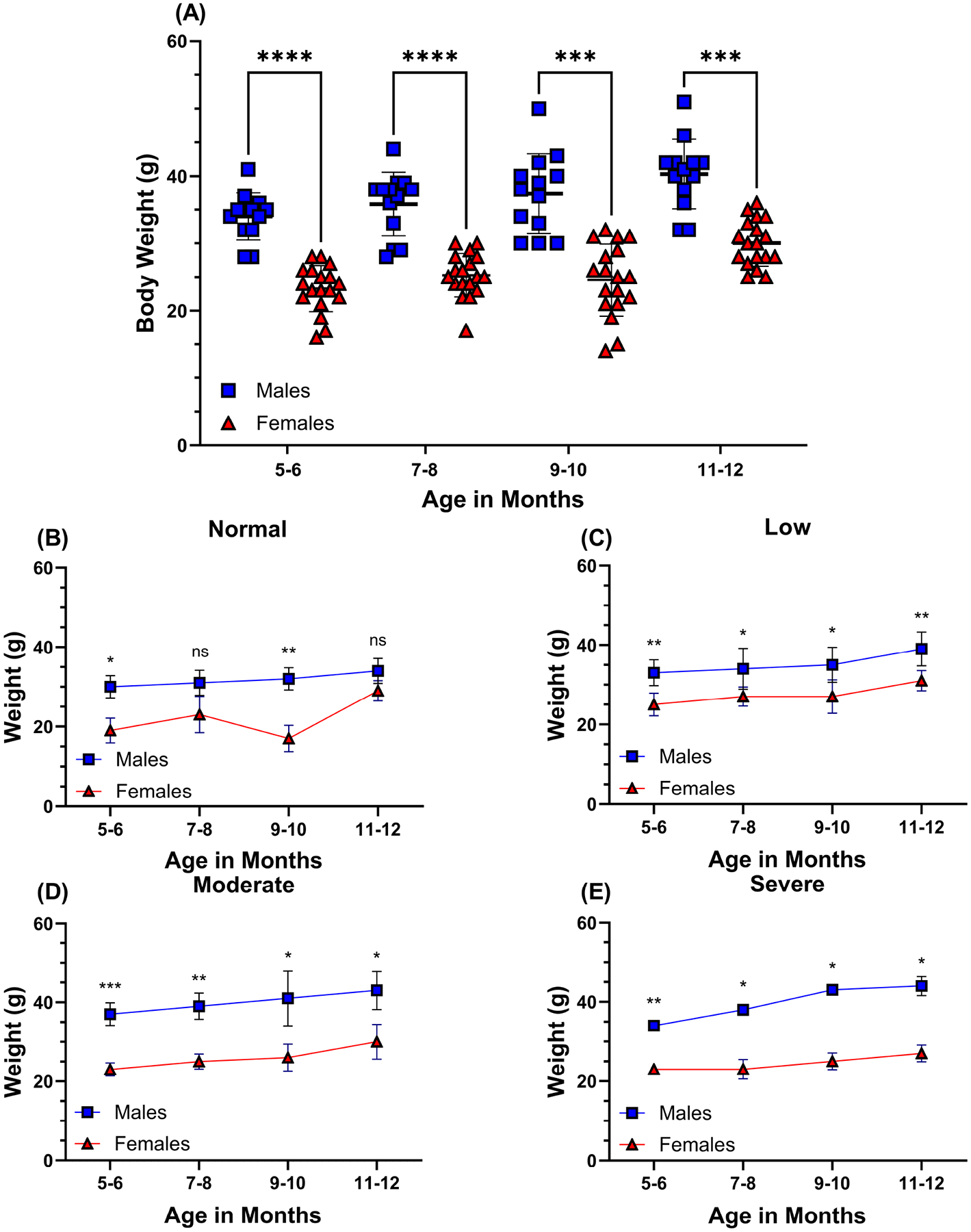
Body weight differences of aged-matched male and female C57BL/6J mice. The data presents monthly measurements of mean body weights (in grams) recorded for all mice throughout the study (A). Smaller representative graphs indicate mean body weights for male and female mice eyes stratified according to the degree of vision loss: normal vision (n = 7), low (n = 18), moderate (n = 15), and severe vision loss (n = 8), determined by behavior assessment of optomotor reflex (B–E). The visual acuity scale is shown in Fig. 2B. Male mice (n = 13) are symbolized by blue squares, and female mice (n = 18) are symbolized by red triangles. Results are mean ± SD. Two-tailed, unpaired *t*-tests (A–E) were used to determine the statistical significance of mean values for each group of mice. *, **, *** and **** designate statistically significant differences (*p* < 0.05, *p* < 0.01, *p* < 0.001, and *p* < 0.0001) between mean values. ns, not significant; SD, standard deviation.

We began our study by investigating whether the observed weight differences between male and female mice were associated with corresponding disparities in visual acuity impairment. In this study, animals were weighed before each VA assessment. As shown in Fig. 1, male C57BL/6J mice displayed an overall statistically significant increase in body weight compared to age-matched females throughout this study (Fig. 1A) [55]. At 5 months, male mice initially weighed 34 g ± 4, while female mice averaged 24 g ± 2, *p* < 0.0001. The final weight at the end of the study for male mice was 40 g ± 5, while for female mice was 30 g ± 3, *p* < 0.001, a difference of 6 g for both sexes from the beginning of the study.

### 3.2 Decline in the Visual Function of Male and Female, Age-Matched C57BL/6J Mice

**Fig. 2.**
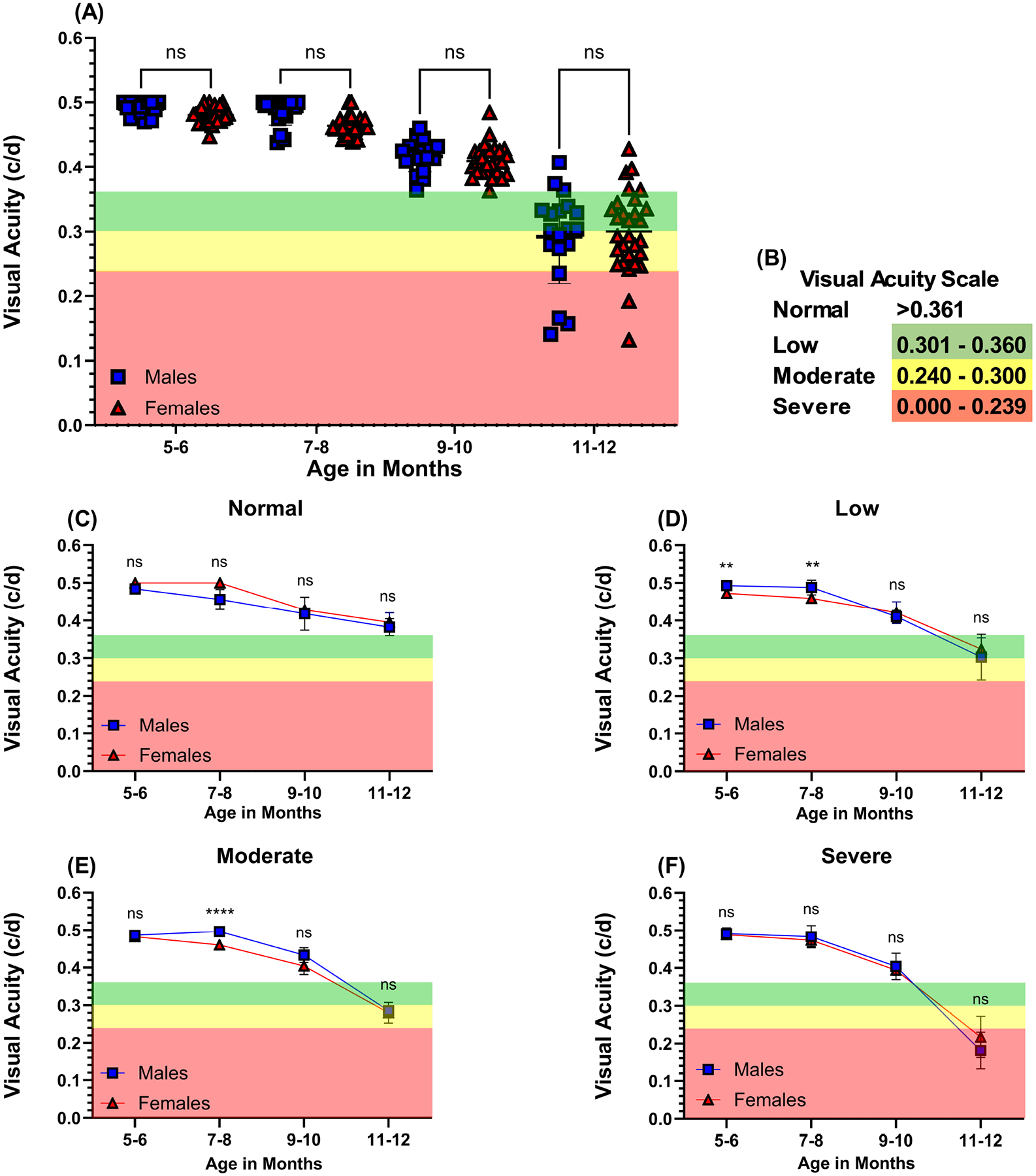
Visual acuity in middle-aged C57BL/6J mice. Visual acuity (A) was assessed in mice monthly by behavior assessment of optomotor reflex expressed as spatial frequency units, cycles/degree (c/d). Blue squares symbolize individual male mice eyes (n = 19), and red triangles symbolize individual female mice eyes (n = 29). Scale for the severity of visual loss expressed by spatial frequency (c/d) is normal (n = 7; white) >0.361, low (n = 18; green) 0.360–0.301, moderate (n = 15; yellow) 0.300–0.240, and severe (n = 8; red) 0.239–0.000 (B). Smaller representative graphs indicate differences between age-matched males in blue and females in red according to the degree of vision loss determined by behavior assessment of optomotor reflex (C–F). Data are presented as mean ± SD. Two-tailed, unpaired *t*-tests (A,C–F) were used to determine the statistical significance of mean values for each group of mice. ** and **** designate statistically significant differences (*p* < 0.01 and *p* < 0.0001) between mean values. ns, not significant.

Visual function (VA and CS) was quantified by behavioral assessment of optomotor reflex (OMR) beginning at 5 months of age (Fig. 2A,B). No statistically significant differences were found in visual function between male and female mice aged 5–12 months. Visual acuity started to decline at 9–10 months of age and declined even further by the study’s conclusion. Male mice’s mean initial VA at 5 months of age was 0.490 (c/d) ± 0.011, while female mice had a mean initial VA of 0.480 (c/d) ± 0.012, *p* > 0.05. The final mean VA at the end of the study was 0.292 (c/d) ± 0.072 for male mice and 0.300 (c/d) ± 0.063, *p* > 0.05 for female mice, a decline of 0.198 (c/d) and 0.180 (c/d), respectively, over the 7-month observation period. At the end of the study, eyes were categorized into groups based on the extent of vision impairment: normal vision (Fig. 2C), low (Fig. 2D), moderate (Fig. 2E), and severe vision loss (Fig. 2F).

**Fig. 3.**
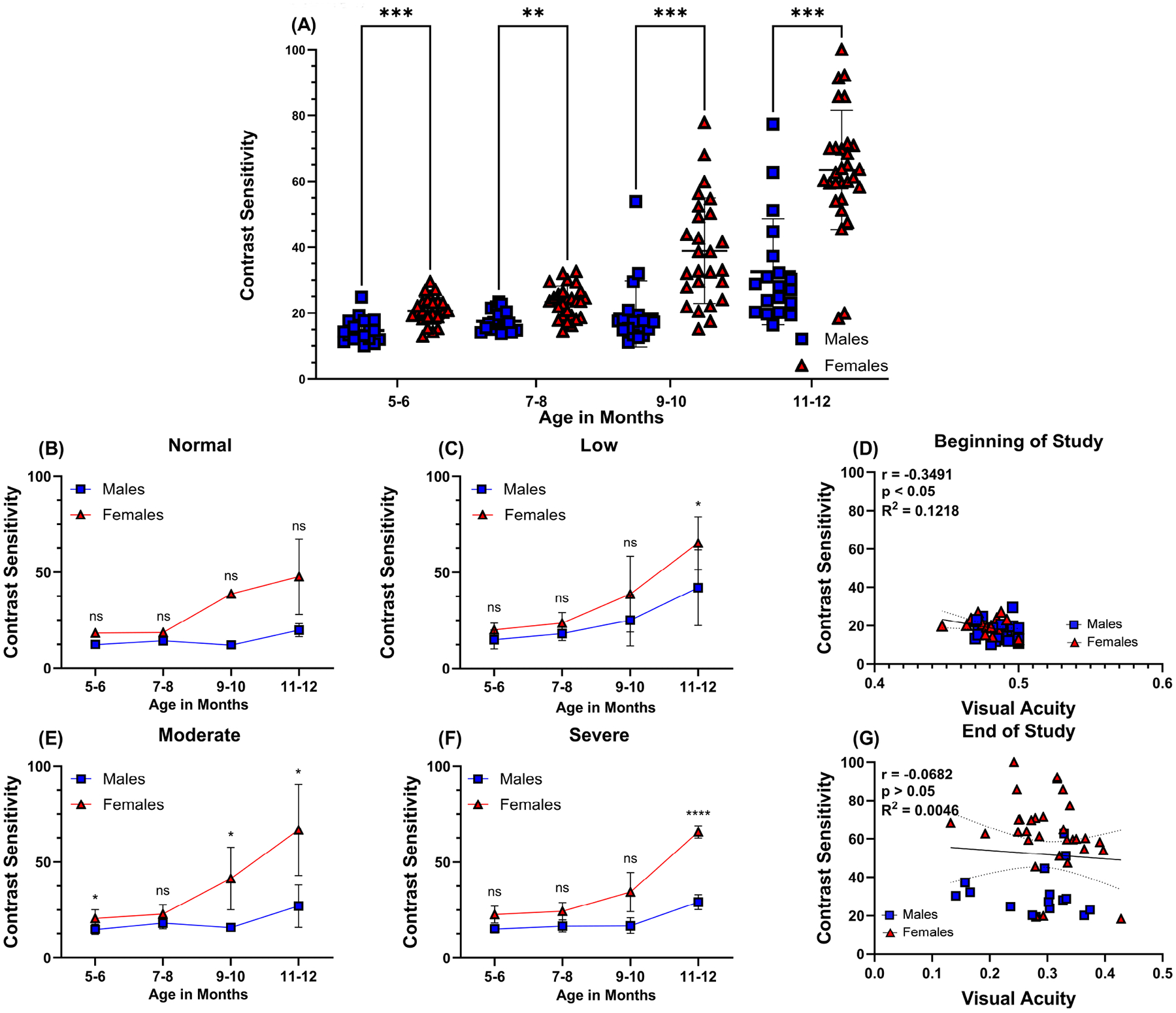
Contrast sensitivity in middle-aged C57BL/6J mice. Contrast sensitivity (A) was assessed in mice monthly by behavior assessment of optomotor reflex. Blue squares symbolize individual male eyes (n = 19), and red triangles symbolize individual female mice eyes (n = 29). Smaller representative graphs (corresponding animals chosen from representative VA graphs from Fig. 2C–F indicate differences between age-matched males in blue and females in red according to the degree of vision loss: normal vision (n = 7), low (n = 18), moderate (n = 15), and severe vision loss (n = 8), determined by behavior assessment of optomotor reflex (B–E). Correlations between VA and CS were determined at the beginning (F) and end of the study (G) for all eyes. Dashed lines around the regression line indicate 95% CI. Specific Pearson correlation coefficient r, respective *p*-values, and coefficient of determination R^2^ are listed directly in panels. Data are presented as mean ± SD. Two-tailed, unpaired *t*-tests were used to determine the statistical significance of mean values for each group of mice (A–C and E,F). *, **, *** and **** designate statistically significant differences (*p* < 0.05, *p* < 0.01, *p* < 0.001, and *p* < 0.0001) between mean values. ns, not significant; VA, visual acuity; CS, contrast sensitivity.

Assessment of CS also identified sex as a biological variable with statistically significant differences between male and female mice (Fig. 3A). Contrast sensitivity started to decline at 9–10 months of age and continued to decline further by the end of the study. The mean initial CS for male mice at 5 months of age was 14.7 ± 3.7, while the mean initial CS for female mice was 20.7 ± 4.0, *p* < 0.001. The final mean CS at the end of the study was 32.5 ± 16.1 for male mice and 63.4 ± 18.1, *p* < 0.001 for female mice, a decline in contrast 17.8 and 42.7, respectively, from the beginning of the study. A higher mean CS percentage indicates less sensitivity to fine detail and moving stimuli in female mice measured at 12 months of age. Data collected from OMR were stratified according to the degree of vision loss: normal vision (Fig. 3B), low (Fig. 3C), moderate (Fig. 3D), and severe vision loss (Fig. 3E). We also examined the relationship between VA and CS at the beginning (Fig. 3F) and end of the study (Fig. 3G). There was a weak, statistically significant, negative correlation between VA and CS at the beginning of the study (r = −0.3491, *p* < 0.05, R^2^ = 0.1218), but by the end of the study, there was no correlation (r = −0.0682, *p* > 0.05, R^2^ = 0.0046), indicating that aging affects these two aspects of visual function with no apparent pattern.

### 3.3 Differences in the ONL Thickness Quantified from in Vivo OCT Imaging

**Fig. 4.**
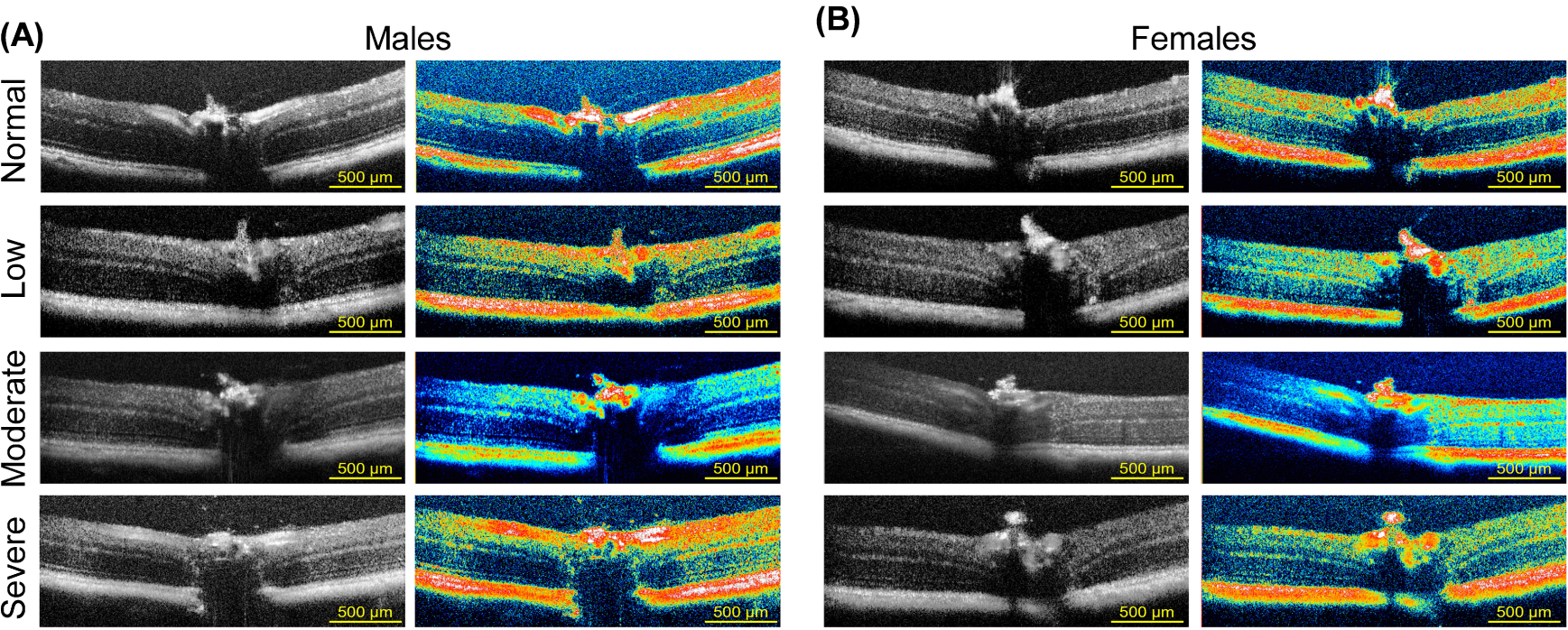
*In vivo* SD-OCT imaging in middle-aged C57BL/6J mice. Representative OCT B-scan images demonstrating retinal structure (black and white images) and corresponding royal pseudo color to enhance the contrast between retinal layers in male (A) and age-matched female mice (B). Images were also stratified according to the degree of vision loss: normal vision, low, moderate, and severe vision loss determined by behavior assessment of optomotor reflex. Scale bar = 500 μm. OCT, optical coherence tomography; SD-OCT, Spectral-domain-OCT.

Horizontal cross-sections of retinas from male (Fig. 4A) and age-matched female (Fig. 4B) C57BL/6J mice were imaged by SD-OCT. Images are shown in black and white and pseudo-colored to visualize the reflective signal’s intensity better, allowing for greater contrast between individual retinal layers. Layers colored red to white indicate tissues with high reflectivity as opposed to layers colored green to yellow. Representative images are grouped according to the degree of visual impairment using spatial frequency OMR stratification described above (normal vision, low, moderate, and severe vision loss). Images in Supplementary Fig. 1 identify and indicate the labeling of layers for quantification purposes using a 5-month-old C57BL/6J female mouse retina (Supplementary Fig. 1A) compared to a 15-month-old C57BL/6J female mouse retina (Supplementary Fig. 1C). No differences in thickness were noted between the nasal and temporal retina to the optic nerve (data not shown). Representative OCT images were arranged to visualize differences between male and female retinas belonging to 5-month-old animals (Supplementary Fig. 1B) and 15-month-old male (Supplementary Fig. 1D) and female (Supplementary Fig. 1E) mouse retinas stratified according to the degree of visual impairment. The ONL contains the cell bodies of rod and cone photoreceptors. The structure of the ONL serves as an essential biomarker of retina neurodegeneration [56,57]. ONL thinning can be seen in the representative images for low, moderate, and severe vision loss compared to normal vision, though more pronounced in male mice (Supplementary Fig. 1D).

**Fig. 5.**
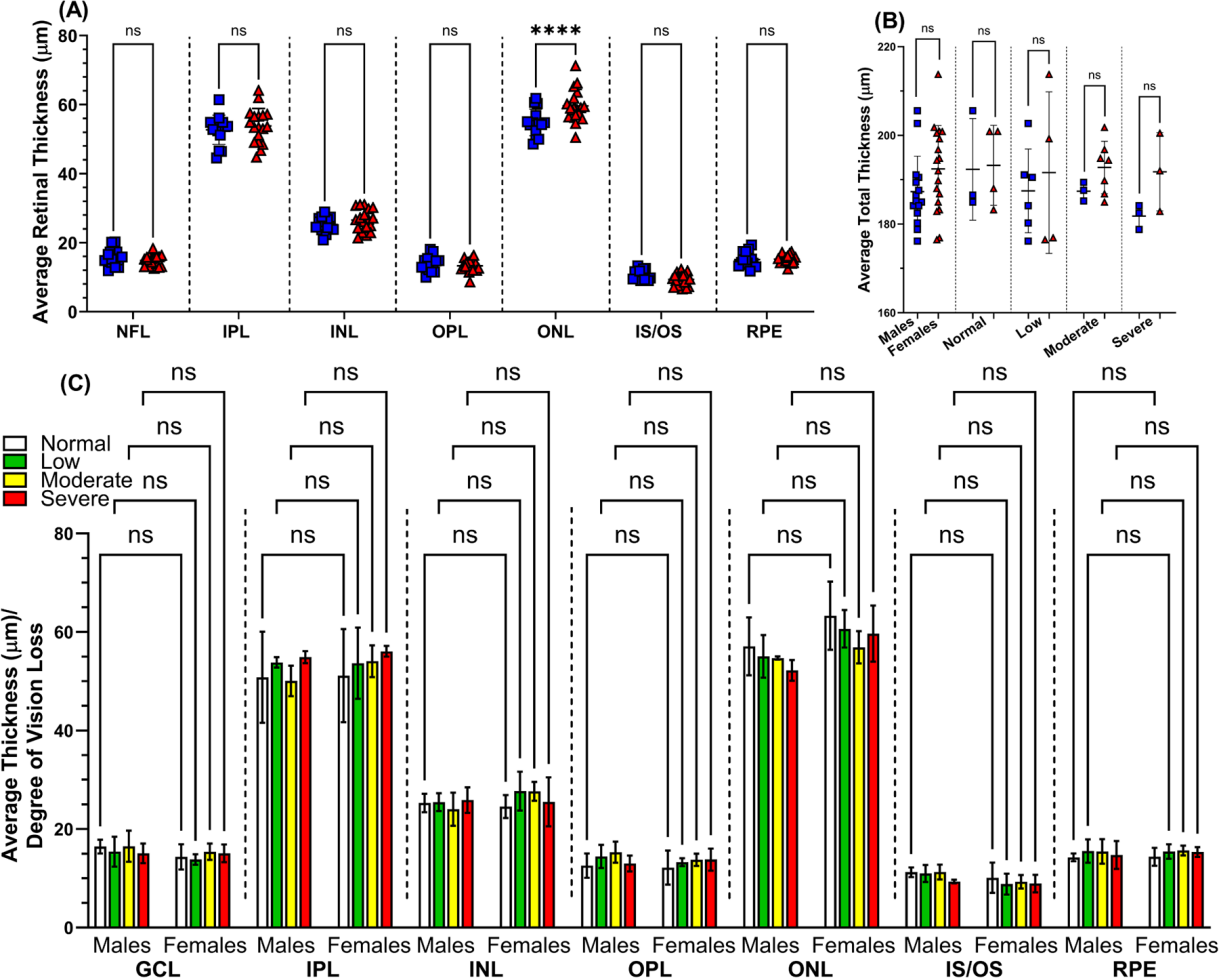
Quantification of retinal layer thickness from optical coherence tomography imaging. OCT B-scan images were manually reviewed and segmented, and each layer was measured for thickness by comparing male and female mice using Fiji – ImageJ processing software (A). The mean total retinal thickness for male and female mice was stratified according to the degree of vision loss: normal vision (n = 7), low (n = 18), moderate (n = 15), and severe (n = 8) vision loss determined by behavior assessment of optomotor reflex (B). Blue squares symbolize male mice (n = 13), and red triangles symbolize female mice (n = 18). The mean thickness of individual retinal layers for male and female mice was also categorized according to the degree of vision loss determined by behavior assessment of optomotor reflex (C). Data are presented as mean ± SD. Two-tailed, unpaired *t*-tests (A,B) were used to determine the statistical significance of mean values for each group of mice. Two-way ANOVA with Tukey’s post-hoc test was used to determine the statistical significance of mean values for each group of mice in C. **** designates a statistically significant difference (*p* < 0.0001) between mean values. ns, not significant; NFL, nerve fiber layer; INL, inner nuclear layer; ONL, outer nuclear layer; IPL, inner plexiform layer; IS, inner segment; OS, outer segment; GCL, ganglion cell layer; OPL, outer plexiform layer; RPE, retinal pigment epithelial.

Individual retinal layer thicknesses were quantified from SD-OCT scans as detailed above, with the ONL in male mice showing a statistically significant decrease in thickness compared to female mice (54.83 μm ± 3.85 and 59.63 μm ± 4.73, *p* < 0.0001, respectively) suggesting that ONL thickness decreases differentially over time between sexes (Fig. 5A). The mean total retinal thickness was not statistically different between male and age-matched female mice (Fig. 5B) (187.30 μm ± 8.02 and 192.45 μm ± 9.74, *p* > 0.05, respectively). In addition, the mean total retinal thickness for each visual impairment group did not show statistical significance between male and female mice: normal vision (192.34 μm ± 11.47 and 190.70 μm ± 9.03, *p* > 0.05, respectively), low (187.48 μm ± 9.44 and 191.59 μm ± 18.22, *p* > 0.05, respectively), moderate (187.41 μm ± 2.11 and 192.77 μm ± 5.91, *p* > 0.05, respectively), and severe vision loss (181.79 μm ± 2.73 and 191.79 μm ± 8.83, *p* > 0.05, respectively) (Fig. 5B). The mean thickness of each retinal layer was also stratified by the degree of vision loss measured from each eye: normal vision (white), low (green), moderate (yellow), and severe vision loss (red) (Fig. 5C). There were no statistically significant differences in mean retinal layer thicknesses found between male and female mice when stratified by degree of vision loss (Fig. 5C).

Pearson correlations were performed for visual function variables, VA and CS, to total retinal thickness (Supplementary Fig. 2A,B), INL thickness (Supplementary Fig. 2C,D), and ONL thickness (Supplementary Fig. 2E,F) quantified from OCT images of each eye at the end of the study, to test for consistency in the data. No appreciable correlation was found for VA to total retinal thickness, INL thickness, and ONL thickness in male and female mice. However, CS for C57BL/6J male mice to total retinal thickness (Supplementary Fig. 2A) showed a moderate, negative correlation that was statistically significant (r = −0.5318, *p* < 0.05, R^2^ = 0.2828).

### 3.4 Significant Differences in Electroretinogram Responses between Aged-Matched Male and Female Mice

**Fig. 6.**
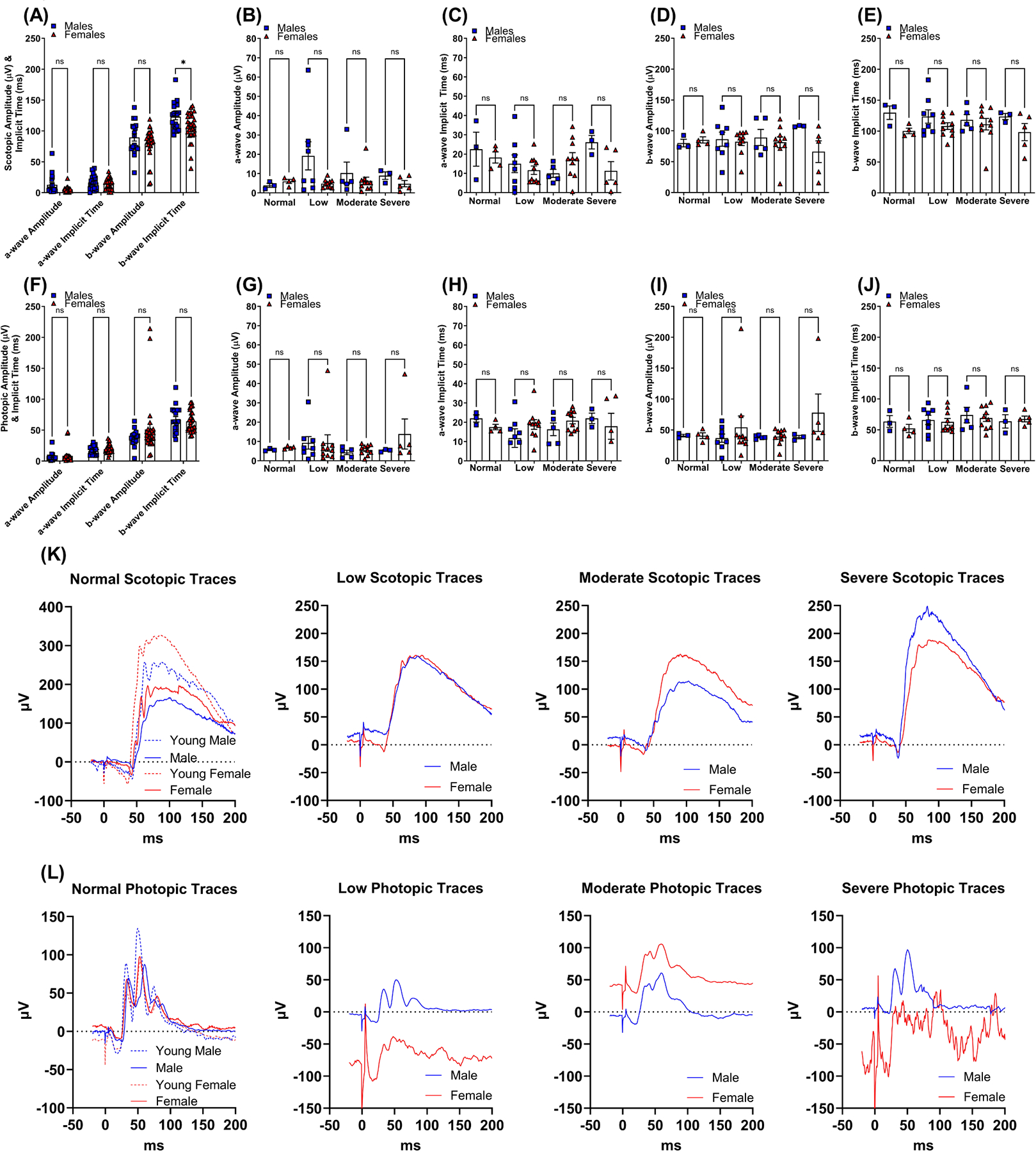
Retinal function differences determined by electroretinogram of middle-aged C57BL/6J mice. Full-field flash ERG was used to determine scotopic and photopic amplitude (A,B,D and F,G,I, respectively) and implicit time (A,C,E and F,H,J, respectively) in 15-month-old age-matched male mice eyes (n = 19) represented by blue squares and female mice eyes (n = 29) represented by red triangles. Scotopic and photopic ERG responses are categorized into normal vision (n = 7), low (n = 18), moderate (n = 15), and severe (n = 8) vision loss (B–E,K, and G–J,L, respectively) determined by behavior assessment of optomotor reflex. Mean representative line graphs indicate ERG differences between males in blue and females in red according to the degree of vision loss. Individual male eyes (n = 10) and individual female eyes (n = 8) of 5-month-old C57BL/6J mice were used as comparisons (open blue squares with dashed lines and open red triangles with dashed lines). Data are presented as mean ± standard error of mean (SEM). Two-tailed, unpaired *t*-tests (A–E and F–J) were used to determine the statistical significance of mean values for each group of mice. * designates a statistically significant difference (*p* < 0.05) between mean values. ns, not significant; ERG, electroretinogram.

Full flash ERG measurements of scotopic (Fig. 6A–E,K) and photopic (Fig. 6F–J,L) functions were recorded from aged-matched male and female mice. Delays in mean ERG responses for scotopic b-wave implicit time from male mice were statistically significant compared to female mice (123.2 ms ± 5.2 and 106.4 ms ± 4.3, *p* < 0.05, respectively, Fig. 6A). Eyes were also stratified according to the degree of vision loss using spatial frequency OMR responses described above (normal vision, low, moderate, and severe vision loss; Fig. 6B–E,G–J). No statistically significant differences were found in scotopic and photopic amplitudes and implicit times when stratified by degree of vision loss (Fig. 6K,L). However, mean photopic responses for low, moderate, and severe vision loss displayed differences between sexes with increased visual impairment (Fig. 6L). Middle-aged animals displayed a decline in amplitude and delay in implicit time compared to young animals (3–5 months old) for the scotopic and photopic retinal function shown in the lower panels (Fig. 6K,L). These findings suggest that sex as a biological variable in electroretinography is a confounding factor for implicit time and photopic function when testing mixed-sex cohorts. In planning and executing ERG experimental studies, it is essential to account for age and sex as significant biological factors.

Single flash recordings for scotopic (Supplementary Fig. 3A–D) and photopic (Supplementary Fig. 3E–H) amplitude and implicit time show differences between male and female mice. Individual flashes for scotopic b-wave implicit time (Supplementary Fig. 3D) at −5.5 log cd·s/m^2^ (*p* < 0.01) showed statistically significant differences in female compared to male mice. Photopic recordings with a statistically significant difference include photopic b-wave amplitude (Supplementary Fig. 3G) 1.0 log cd·s/m^2^ (*p* < 0.05). The results from our ERG analyses exhibit a small number of statistically significant differences from single flashes for scotopic and photopic protocols. Differences in amplitude (Supplementary Fig. 3G) were found to occur midway through the protocol, while implicit time delays (Supplementary Fig. 3D) happened at the beginning of the protocol.

**Fig. 7.**
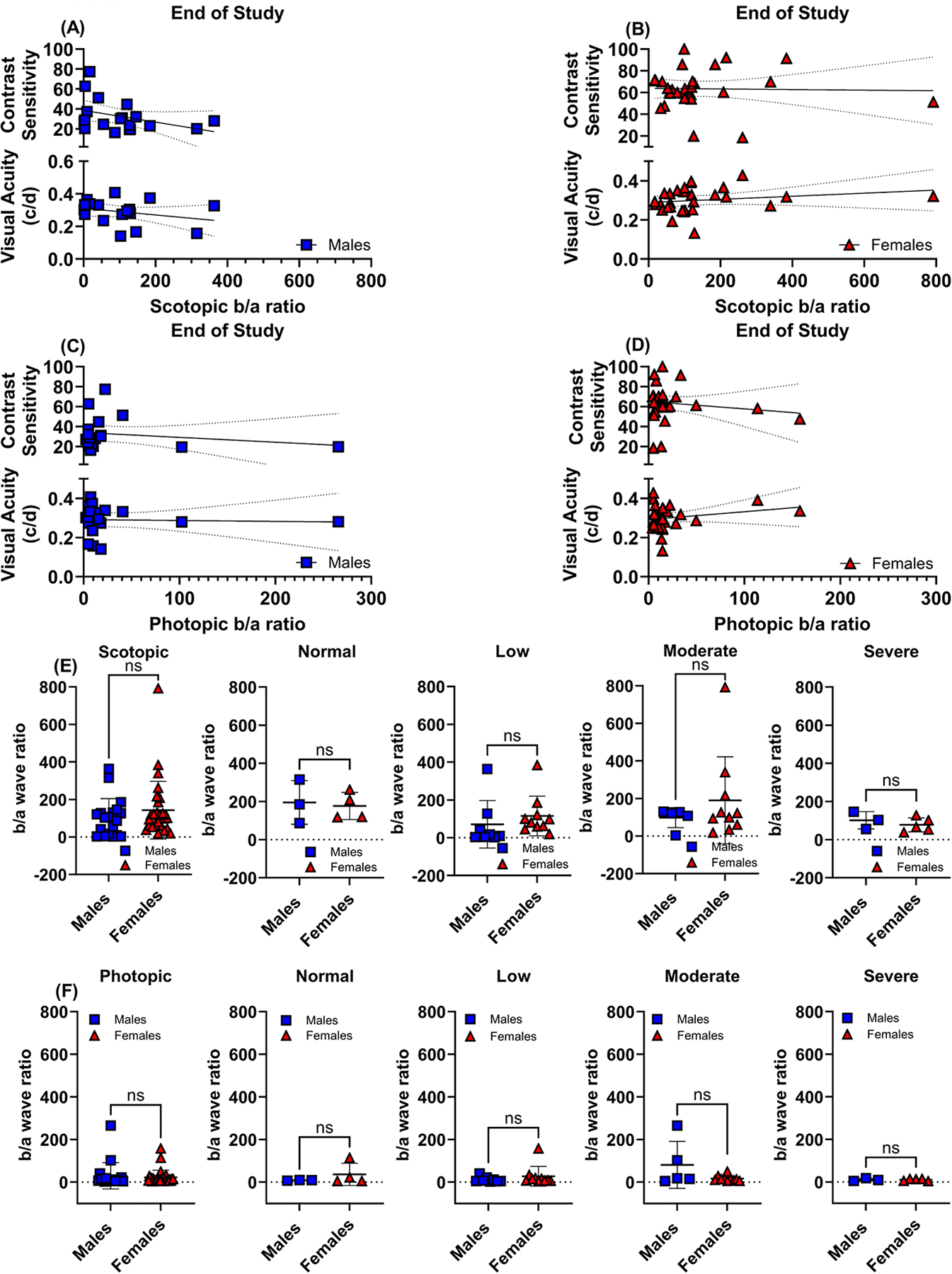
Relationship between visual function and retinal function represented by ERG b/a ratio. Correlations were determined for male and female C57BL/6J mice eyes between behavior assessment of visual function (VA and CS) and scotopic (A,B, respectively) and photopic (C,D, respectively) b/a amplitude ratios calculated at the end of the study. Dashed lines around the regression line indicate 95% CI. Pearson correlation coefficient r, *p*-values, and goodness of fit R^2^ are listed directly in panels where significance was found. The mean amplitudes corresponding to the ERG b/a wave ratio between age-matched male and female mice were calculated for scotopic (E) and photopic (F) responses and stratified according to visual impairment: normal vision (n = 7), low (n = 18), moderate (n = 15), and severe (n = 8) vision loss. Blue squares symbolize male mice’s eyes (n = 19), and red triangles symbolize female mice’s eyes (n = 29). Data are presented as mean ± SD. Two-tailed, unpaired *t*-tests (E,F) were used to determine the statistical significance of mean values for each group of mice. ns, not significant.

The b/a wave ERG ratio is a predictive measure for assessing the functional relationship between the inner and outer layers of the retina. Pearson correlation analyses were conducted to evaluate the association between VA and CS with the scotopic b/a wave ratios (refer to Fig. 7A,B) and the photopic b/a wave ratios (see Fig. 7C,D) at the conclusion of the study. No correlations were found between VA and CS with b/a wave ERG ratios. Neither scotopic b/a wave ratios (Fig. 7E) nor photopic b/a wave ratios (Fig. 7F) exhibited appreciable statistical significance between age-matched male and female mice when eyes were stratified by degree of visual impairment.

Components of scotopic threshold responses (STRs) (Supplementary Fig. 4A–D) and photopic threshold responses (PhNRs) (Supplementary Fig. 4E,F) showed statistically significant differences in middle-aged animals compared to young animals for pSTR, STR amplitude, and PhNR latency and young female mice compared to young male mice for STR amplitude (Supplementary Table 1). Oscillatory potential (OP 1–4) amplitudes (Supplementary Fig. 5B,D,F,H) and latency (Supplementary Fig. 5A,C,E,G), thought to be generated by inner retinal neurons [58], showed statistically significant differences with 15-month-old female mice compared to age-matched male mice and for middle-aged animals compared to young animals for flash intensities −2.0 and −1.5 cd·s/m^2^. For OP 1, there were significant differences in amplitude that depended on age, but only at a flash intensity of −1.5 log cd·s/m^2^. However, no significant differences were found in older animals for OP 1 that were age- or sex-dependent. For OPs 2–4, significant differences were found in amplitude and latency depending on age and sex, specifically at flash intensities of −2.0 and −1.5 log cd·s/m^2^ (Supplementary Table 2).

### 3.5 Differences in the Retinal Layer Thickness Quantified from Histological Analysis

**Fig. 8.**
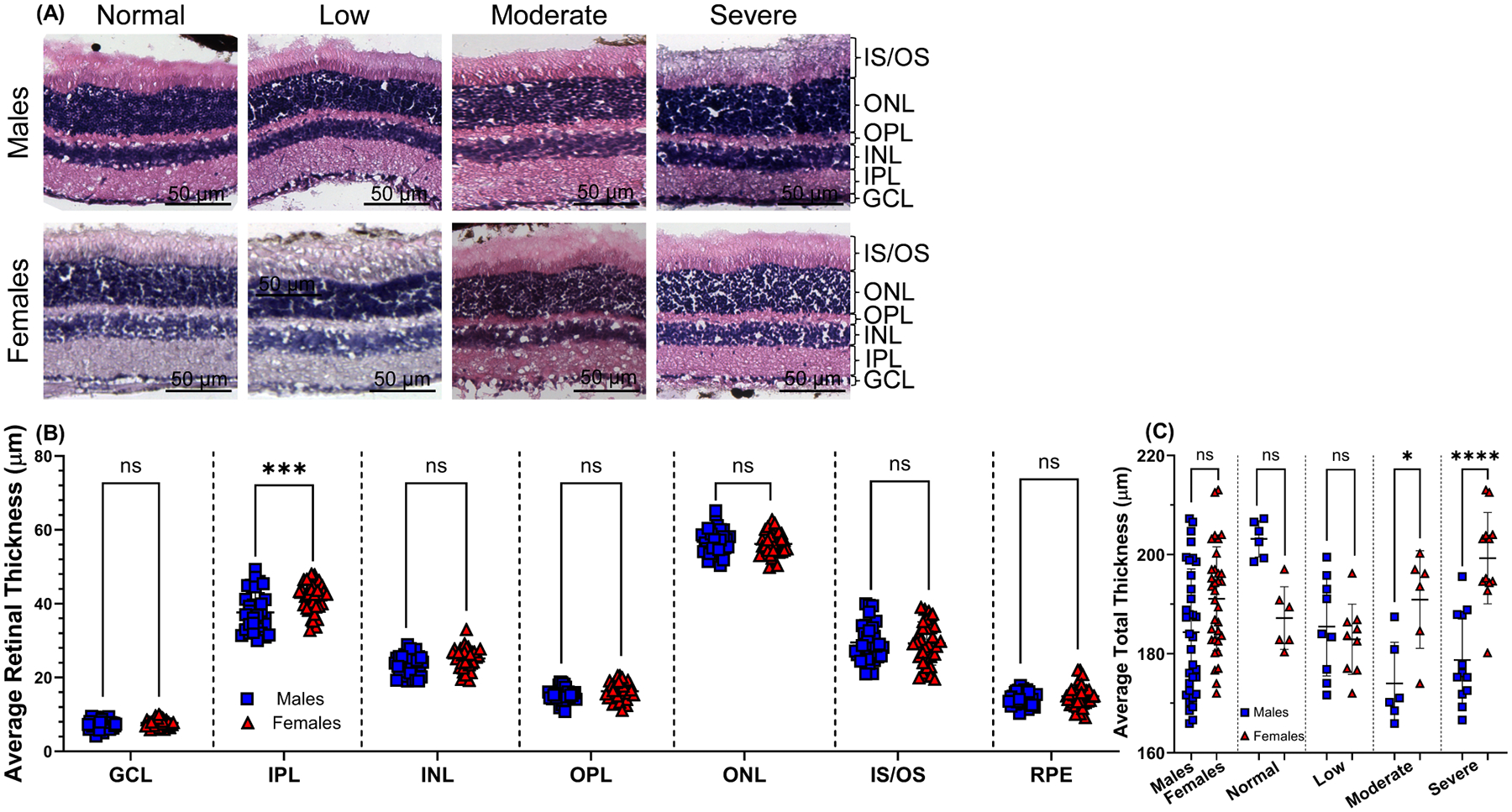
Differences in retinal morphology of middle-aged C57BL/6J mice. Representative retinal sections comparing each category of vision loss for 15-month-old males to age-matched female mice eyes and stratified according to the degree of visual function loss (A). Quantification for individual mean retinal layer thickness for male (n = 11) and age-matched female (n = 11) mice eyes (B). The mean total retinal thickness for male and female mice eyes was also determined and stratified depending on visual impairment: normal vision (n = 6/group), low (n = 9/group), moderate (n = 6/group), and severe vision loss (n = 4/group) (C). Blue squares symbolize male mice eyes, and red triangles symbolize female mice eyes. Data are presented as mean ± SD with three measurements/eye. Two-tailed, unpaired *t*-tests (B,C) were used to determine the statistical significance of mean values for each group of mice. *, ***, **** designates a statistically significant difference (*p* < 0.05, *p* < 0.001, and *p* < 0.0001) between mean values. H&E, 20× magnification, scale bar = 50 μm. ns, not significant; H&E, hematoxylin and eosin staining.

Histological studies on retinal tissue sections stained with H&E for male (n = 11) and age-matched female mice (n = 11), stratified according to the degree of vision loss: normal (n = 2), low (n = 3), moderate (n = 2), and severe (n = 4) for each sex, are shown by representative images in Fig. 8A. A detailed overview of retinal layers representing visual impairment shows overall gross changes in morphology compared to retinal layers representing normal vision. Histological analysis demonstrated that the IPL for male mice was significantly thinner than for female mice (37.63 μm ± 5.42 and 41.57 μm ± 4.14, *p* < 0.001, respectively). As the visual function deteriorated, noticeable alterations were observed. Variations in the thickness and structure of the IPL, INL, ONL, and photoreceptor inner segment/outer segment (IS/OS) layers were identified between male and female mice (Fig. 8B). The mean total retinal thickness was not statistically different between male and age-matched female mice (Fig. 8C) (184.34 μm ± 12.75 and 191.104 μm ± 10.48, *p* > 0.05, respectively). Interestingly, the mean total retinal thickness for visual impairment groups moderate and severe vision loss displayed statistically significant differences: normal vision (203.17 μm ± 3.66 and 187.20 μm ± 6.32, *p* > 0.05, respectively), low (185.47 μm ± 9.46 and 182.91 μm ± 7.09, *p* > 0.05, respectively), moderate (174.00 μm ± 8.32 and 190.92 μm ± 5.91, *p* < 0.05, respectively), and severe vision loss (178.73 μm ± 9.08 and 199.28 μm ± 9.21, *p* < 0.0001, respectively) (Fig. 8C). As visual impairment worsens, male mice are differently affected by loss of retinal layer thickness compared to females.

**Fig. 9.**
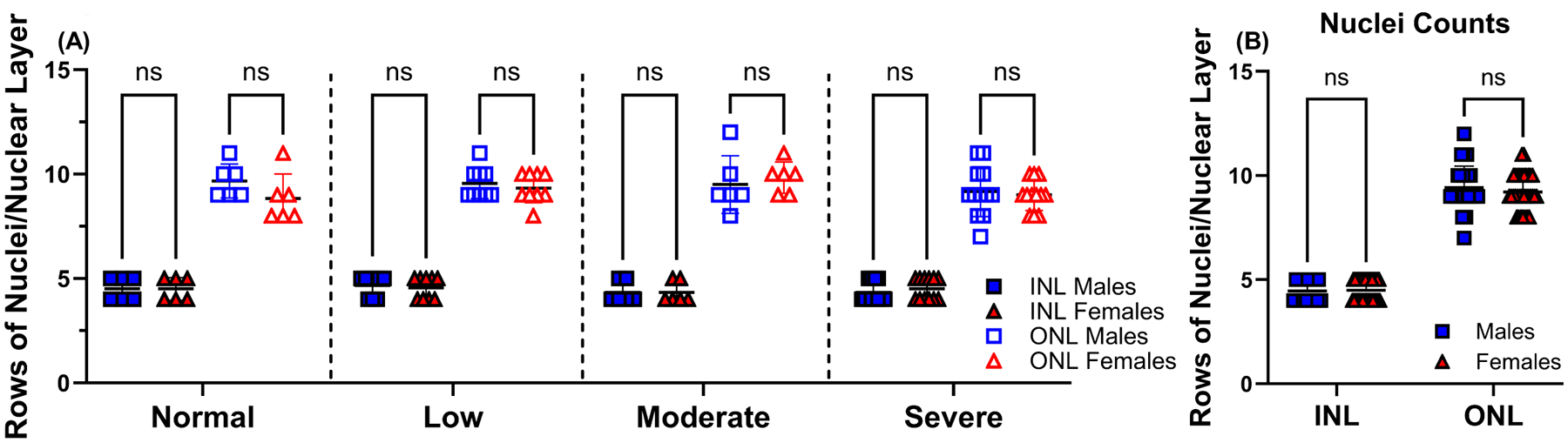
The number of nuclei rows in the INL and ONL does not vary depending on visual impairment. Rows of nuclei in the INL (filled symbols) and ONL (open symbols) were counted for 15-month-old male (n = 11) and age-matched female (n = 11) mice eyes and stratified according to the degree of vision loss: normal vision (n = 6/group), low (n = 9/group), moderate (n = 6/group), and severe vision loss (n = 4/group), determined by behavior assessment of optomotor reflex for 15-month-old male (blue squares) and female (red triangles) C57BL/6J mice (A). Rows of nuclei in the INL and ONL were counted for 15-month-old males (n = 11), represented as blue squares, and females (n = 11), represented as red triangles (B). Data are presented as mean ± SD with three measurements/eye. Two-tailed, unpaired *t*-tests (A,B) were used to determine the statistical significance of mean values for each group of mice. ns, not significant.

Rows of nuclei that make up the INL and ONL were counted, and the resulting data was stratified based on the severity of vision loss using VA testing as described above (normal vision, low, moderate, and severe vision loss; Fig. 9A). No statistically significant differences were noted in the ONL and INL of male and female retinas. Overall, the mean number of nuclei rows counted in both layers from middle-aged male mice decreased as vision became more impaired. No discernible pattern of nuclei row loss was observed in female mice. Counts of nuclei rows of INL and ONL that were not stratified according to visual impairment showed no significant differences between male and female retinas (4.45 ± 0.51, 9.42 ± 1.03, *p* > 0.05, and 4.48 ± 0.51, 9.21 ± 0.86, *p* > 0.05, respectively) (Fig. 9B).

**Fig. 10.**
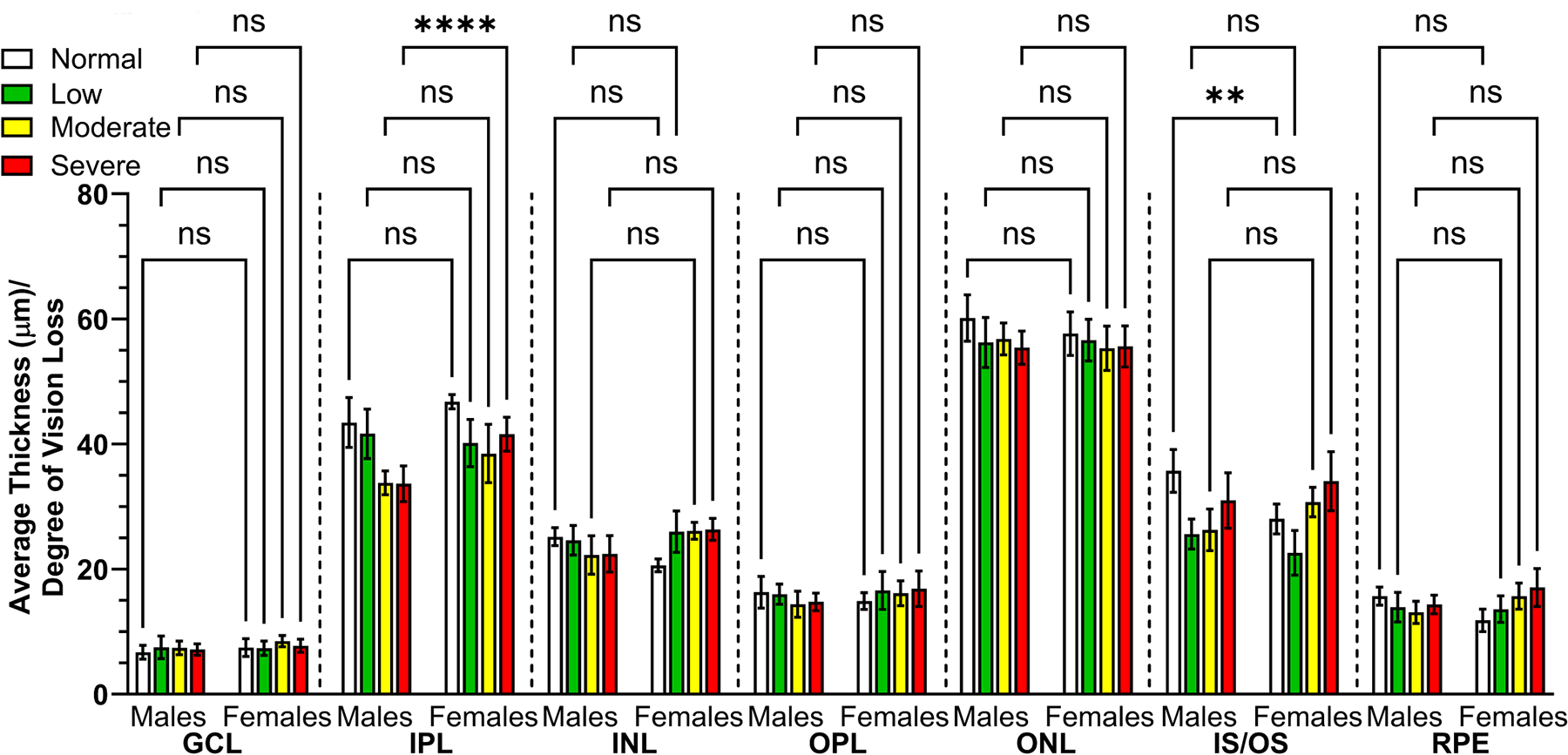
Disparities observed in the mean thicknesses of the IPL and IS/OS retinal layers after stratification. The mean thickness of individual retinal layers for 15-month-old male (n = 11) and age-matched female (n = 11) mice eyes were stratified according to the degree of vision loss: normal vision (n = 6/group; white), low (n = 9/group; green), moderate (n = 6/group; yellow), and severe vision loss (n = 4/group; red), determined by behavior assessment of optomotor reflex. Data are presented as mean ± SD with three measurements/eye. Two-way ANOVA with Tukey’s post-hoc test was used to determine the statistical significance of mean values for each group of mice. ** and **** designate a statistically significant difference (*p* < 0.01 and *p* < 0.0001) between mean values. ns, not significant.

Statistically significant sex-specific differences were found in the mean thickness of the IPL in mice as visual impairment worsened, with male mice displaying the most significant decrease in IPL thickness with severe vision impairment (33.65 μm ± 2.86 and 41.58 μm ± 2.73, *p* < 0.0001, respectively; Fig. 10). The IPL contains synapses between bipolar, amacrine, and ganglion cells. The IPL is further divided into ON and OFF bipolar cell axon terminations. The process of motion perception, such as detection and tracking and changes in brightness and hue, begins in the IPL employed by the ON/OFF channels [59]. With respect to this study, sex differences also describing the thinning of the photoreceptor IS/OS layer were noticeably more pronounced in female mice compared to males with normal vision (28.02 μm ± 2.38 and 35.71 μm ± 3.43, *p* < 0.01, respectively; Fig. 10) suggesting a causal relationship with behavioral assessed CS.

## 4. Discussion

We determined age- and sex-specific differences in visual function and retinal structure in C57BL/6J mice as potential underlying differential aging effects leading to visual loss. To our knowledge, this is the first study to empirically stratify visual impairment based on behavioral measurements of visual performance, identifying age and sex as biological variables and reproducible parameters in the absence of disease.

In healthy mammals, aging is associated with changes in body weight. Body weight and percent body fat generally increase with age due to the accumulation of body fat and free fat mass [60]. These alterations in body composition can be sex or strain-specific. Results from a recent study by Rathod and colleagues suggest that satiation and satiety in the control of energy intake are regulated in a sex and age-dependent manner, with fat mass accumulation more significant and variable in males than females [61]. The association of sex and age-related weight gain of the mice in this study confirms this finding. Generally, physiological and behavioral factors influence rodent health, particularly responses to visual acuity challenges, which may, in turn, affect their weight [62–64]. It is also essential to consider genetic makeup, environmental conditions, and the specific type of visual impairment involved. Further research into the weight disparities observed in male and female mice due to visual impairments could provide deeper insights into the physiological and behavioral nuances that may alter rodents’ responses to deficits in visual function.

Visual acuity refers to the highest spatial frequency of a visual stimulus that can trigger a response. On the other hand, CS starts with a stimulus at 100% contrast and is gradually decreased until a threshold is determined. This threshold is identified by examining the tracking behavior exhibited during the OMR [48]. The lower the contrast percentage, the better an animal can distinguish a stimulus in low-contrast environments, such as dusk, dawn, or dense foliage. This test provides a simple and rapid screening of visual function through behavioral observation of the animal and has been used as a first-line screening of vision in rodents [65–68]. OMR plays a crucial role in maintaining visual stability in mice, and its characteristics are influenced by various factors, including ocular abnormalities and genetic variations in different mouse strains [45–47,69]. Our decision to stratify visual impairment was based on VA instead of CS data. This choice was influenced by the superior resolution for fine detail and the increased reproducibility in VA testing. These findings align with reports from other researchers suggesting that the duration of the test could potentially skew CS measurements and affect animal performance.

Furthermore, it is still unclear if the CS impairments associated with specific eye disorders develop quickly or how these impairments vary based on the nature of the pathology [69,70]. It has been documented in patients that CS deficits may develop at early stages of a specific ocular disease even when VA is relatively normal. Our study has identified several indicators that connect early degenerative changes with CS. Through deep learning, Shamsi and colleagues discovered a significant correlation between the thickness of the human ganglion cell layer, the inner plexiform layer, and CS [71]. *In vivo* imaging by spectral-domain optical coherence tomography displayed a significantly thinner ONL in male mice but no significant layer differences among the vision impairment groups. OCT analysis also identified a significant correlation between total retinal thickness and CS in male mice. Histological examination found the IPL of male mice was significantly thinner than female mice with severe visual impairment. Thinning of the photoreceptor IS/OS layers was noticeably more pronounced in female mice compared to males with normal vision. These findings suggest a causal relationship with behavioral assessed CS. Contrast sensitivity is a crucial aspect of visual perception and a more comprehensive assessment of visual function than acuity [72] that enables us to discern and perceive variations in brightness levels. However, as we undergo changes in our retina related to age and sex, including a decrease in the density of specific retinal layers, our ability to detect spatial patterns with low contrast is affected.

Impairment of retinal function measured by ERG responses was affected by age to the same extent in male and female mice. ERG amplitude is defined as the maximum displacement of the wave evoked by the stimulus intensity, while implicit time is the time between the flash and peak response [73]. We demonstrated that retinal function decreases with age, evidenced by a reduction in a- and b-waves and delays in implicit times. Statistically significant differences in scotopic b-wave implicit time delays were evident in female retinas compared to male retinas but not when visual function is separated by stratification of vision loss. Delays in scotopic and photopic implicit time indicate outer retinal photoreceptor degenerative changes and reflect the rate at which an electrical signal is at maximum amplitude [74]. No significant differences were found in a- and b-wave amplitudes when the biological sex variable was considered. Our results suggest that sex is a considerable confounding variable concerning implicit time, but not for amplitude, and confirm earlier reports of a decline in retinal function with age [75,76]. While we identified age-related impairment of both rod and cone photoreceptor cell-mediated vision, the higher susceptibility of rod photoreceptors to age-mediated decline in function when compared to cones is typically a focus of preclinical research [76].

Structural changes in neurons and CNS tissue are common signs of neurodegeneration [77–79]. SD-OCT enables the non-invasive, cross-sectional visualization of the retina *in vivo* and the identification of morphological differences at high resolution. Histochemistry of vertical retinal tissue sections is a complementary approach for comparative analysis alongside SD-OCT. The average thickness of the retina, as observed through OCT, aligns well with the thickness determined from histological sections, according to our findings. The structure of the ONL, where photoreceptor cell somata and nuclei are located, serves as an essential biomarker of retina neurodegeneration [56,57]. The IPL, in contrast, is the area of the retina where synaptic connections between bipolar cells and dendrites of retinal ganglion cells (RGCs) are found [80]. As visual impairment increased with age, the IPL, ONL, and IS/OS layers displayed the most significant differences between male and female mice, but also among other groups of differently reduced VA. As visual impairment increased with age, the concomitant decrease in IPL thickness also resulted in more pronounced differences between males and females as well as among other groups of differentially reduced VA. Visual function measured by behavior assessment of OMR is partly driven by synaptic signaling in the IPL. Impaired motion perception and tracking in human multiple sclerosis (MS) patients are found to be associated with combined RGC/IPL thinning [81] and are associated with deficits in CS, as mentioned earlier in the discussion. A decrease in synaptic density equates to a reduction in IPL thickness.

Direct comparisons between OCT imaging can influence our results from this study due to factors like acquisition region, layer aggregation, or animal age in addition to the process of histological preparation. Differences between OCT and histology quantification can stem from various factors such as imaging techniques, resolution and detail, quantification methods, sample preparation, and the context of use. These differences underscore the distinct nature of OCT and histology, with each technique providing unique advantages based on the specific context of the results. The differences between female and male mice found here exhibit a consistent trend, even though these differences may not be statistically significant. Our findings lend additional support to the research outcomes of other groups, such as results from a study by Batista *et al*. [82] revealed that total retinal thickness decreases with age, while the thickness of individual retinal layers exhibits varying patterns of change in wild-type C57BL6/129S mice. While the literature consistently reports average total retinal thickness values of approximately 200 μm for wild-type mice, the thicknesses of individual retinal layers or layer aggregates exhibit more significant variability with age. Comparatively, few studies have systematically examined these retinal layers and layer aggregate thicknesses, nor have studies examined how the biological factor of sex affects retinal layer thickness in aging [40,82–84]. Fundamentally, a comparison of retinal tissue thickness by OCT and histological analysis revealed that as mice age, the stratification of visual impairment helps disclose the contribution of sex as a biological variable in our results and is most reliably reflected in thickness changes of the IPL, ONL, and IS/OS layers. Retinal layer thickness in normal eyes generally decreases with age and visual impairment in both sexes. Aging studies in mice and rats have observed significant thinning in the IPL, OPL, INL, and ONL [40,85,86]. In light of this study, stratifying visual function results to understand the contribution of sex further as a biological variable should be considered in experimental planning and evaluating results. The results presented herein of aging mice can serve as a reference for future studies as we advance our understanding of age-related neurodegenerative disease progression.

## 5. Conclusions

In conclusion, the present study provides an essential foundation to further our understanding of age-related pathology’s molecular mechanisms, advance therapy development, and integrate visual performance into both fields. Future work aims to delve deeper into the mechanisms that regulate outcomes associated with slowing the progression of age-related retinal degenerative changes and controlling the therapeutic response. By utilizing the C57BL/6J mouse model and incorporating the additional variable of visual function impairment into the results, we can observe a more accurate estimate of the nature of retinal aging changes alongside differences present from sexual dimorphisms.

## Supplementary Material

Supplementary Materials

## Data Availability

The article/supplementary material encompasses the original contributions from the study. For additional queries, please reach out to the corresponding author.
