# Supplementary material for: Stratification of the Extent of Visual Impairment Identifies Sex-Specific Degenerative Changes in Retinal Structure and Function during Aging
Source: J Integr Neurosci. Author manuscript; Available in PMC 2025 May 20. (PMC12091267; doi:10.31083/JIN25805)
Supplement: Supplementary Materials [file NIHMS2081696-supplement-Supplementary_Materials.pdf]

Supplementary Materials

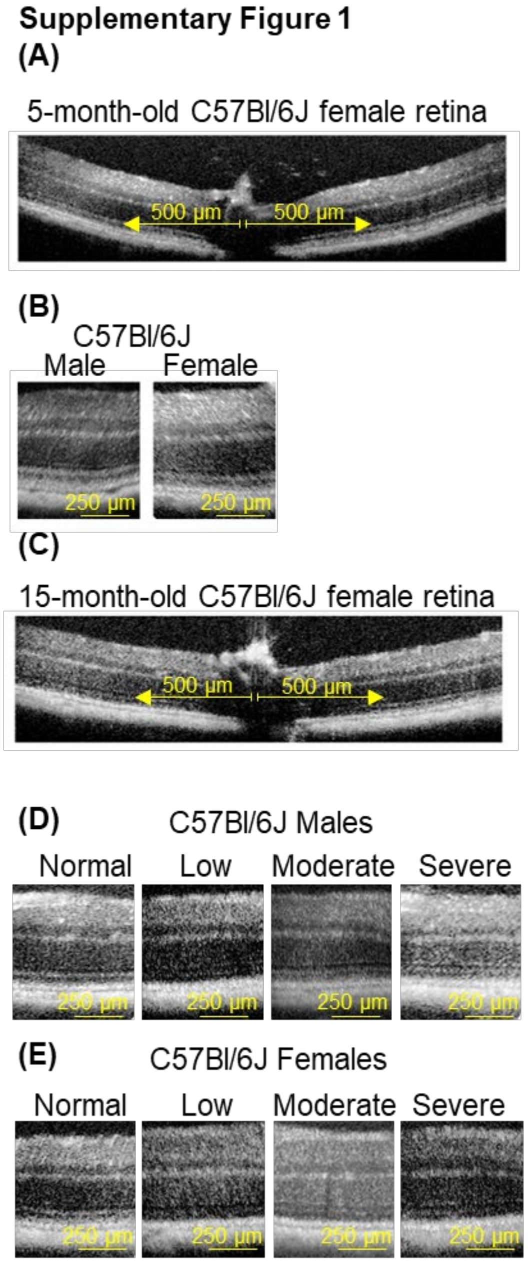

Supplementary Fig. 1: Identification of mouse retinal layers by spectral-domain OCT *in vivo* imaging. Identification of mouse retinal layers in 5-month-old C57Bl/6J female retina (A) and 15-month-old C57Bl/6J female retina (C): retinal nerve fiber layer (NFL), inner plexiform layer (IPL), inner nuclear layer (INL), outer plexiform layer (OPL), outer nuclear layer (ONL), external limiting membrane (ELM), inner segment/outer segment layer (IS/OS), and retinal pigment epithelium (RPE). Layer thicknesses were measured at 500 µm (shown in yellow) from the optic nerve (A and C). Representative sections of OCT images from a 5-month-old male and female mouse indicating 500 microns from the optic nerve were used to visualize and measure differences in total retinal thickness between genders (B). A representative section of an OCT image from each visual category according to the degree of vision loss: normal vision, low, moderate, and severe vision loss, was

used to demonstrate, by quantification, a reduction in total retinal thickness at 15 months of age for male (D) and female (E) mice by the end of the study. Scale = 500 µm for full OCT images (A, C) and 250 µm for stratified OCT images (B, D-E).

Supplementary Figure 2

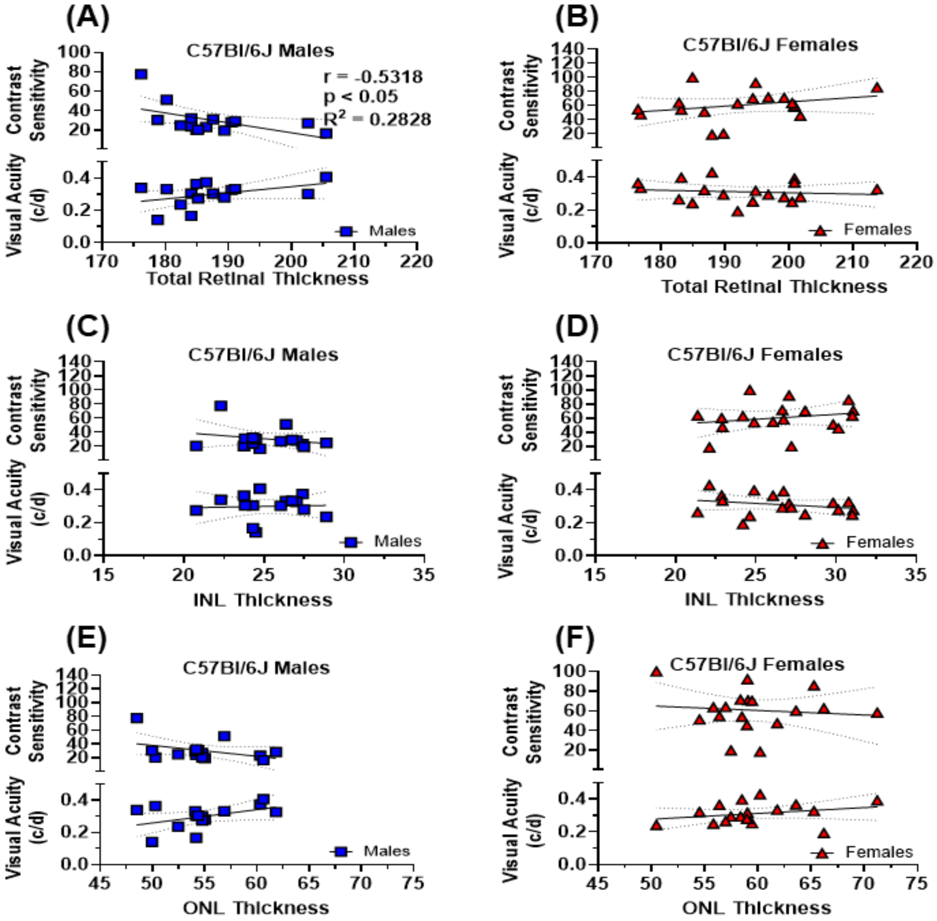

Supplementary Fig. 2: Relationship between visual function and OCT-derived retinal morphology. Pearson correlations were determined for male and female mice between behavior assessment of visual function (VA, lower subpanels, and CS, upper subpanels) and total retinal thickness (A & B), INL thickness (C & D), and ONL thickness (E & F). Male mice eyes (n = 13) are symbolized by blue squares, and female mice eyes (n = 18) with red triangles. In male and female mice, no significant correlation was observed between visual acuity (VA, lower subpanels) and total retinal thickness (A & B), inner nuclear layer (INL) thickness (C & D), or outer nuclear layer (ONL) thickness (E & F). However, in C57Bl/6J male mice, contrast sensitivity (CS, upper subpanels) exhibited a statistically significant moderate negative correlation with total retinal thickness (A). Dashed lines around the regression line indicate 95% CI. Specific Pearson correlation coefficient r, respective p-values, and coefficient of determination R^2 are listed directly in panels where significance is identified.

Supplementary Figure 3

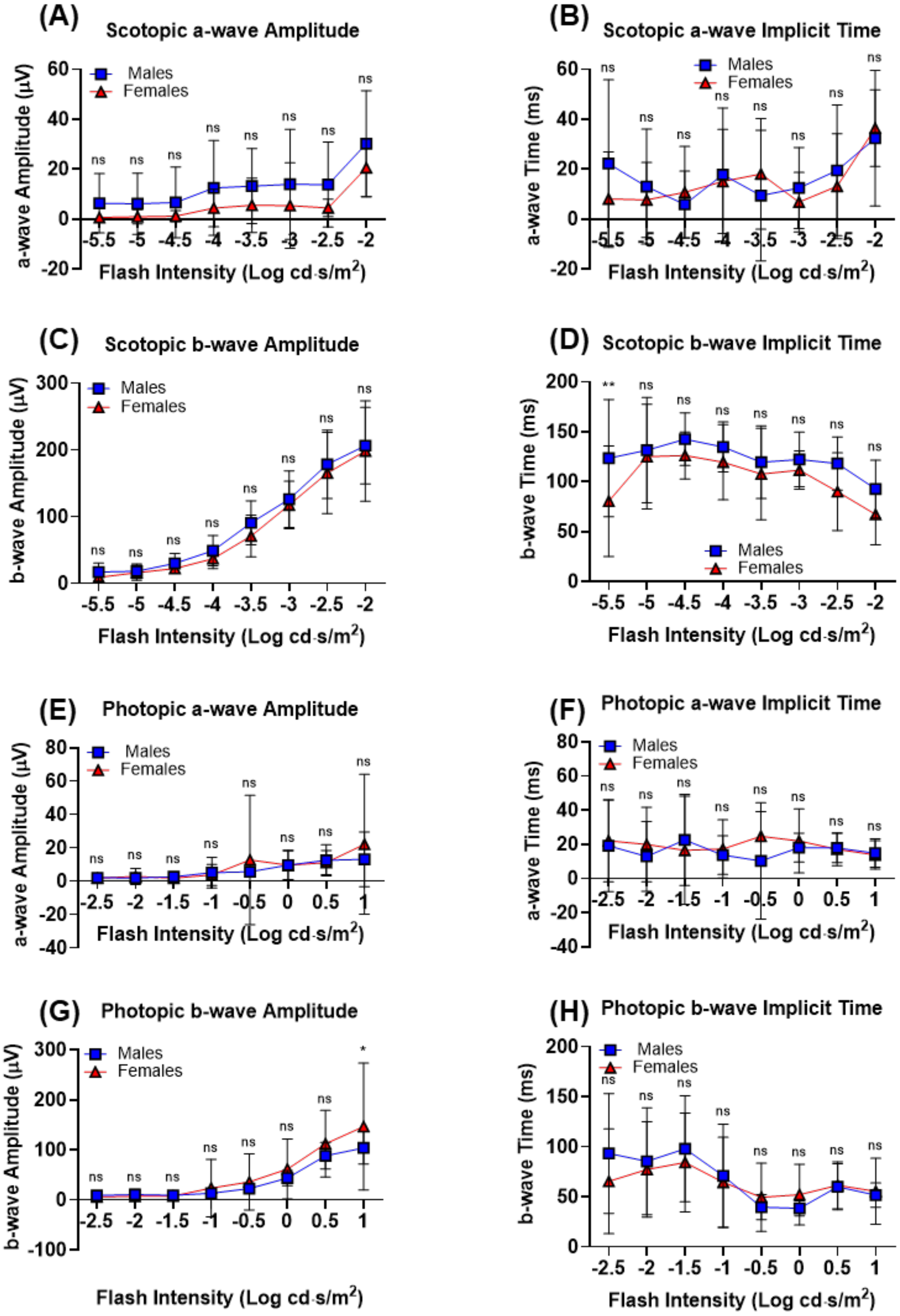

Supplementary Fig. 3: Sex-specific differences in single flash intensities for scotopic and photopic ERG recordings. Scotopic amplitude (A and C), implicit time (B and D), photopic amplitude (E and G), and implicit time (F and H) were measured by full-flash ERG for flash intensities beginning at −5.5 to 1.0 log cd·s/m^2 for the scotopic protocol and -2.5 to 1.0 log cd·s/m^2 for photopic protocol. Lines graphs for individual male mice eyes (n=19) are represented as blue squares with blue lines, while red triangles with red lines represent individual female mice eyes (n=29). Data are expressed as mean ± SEM. Two-way ANOVA with Bonferroni's post-test

was used to determine the statistical significance of mean values for each group of mice. * and ** designate a statistically significant difference ($p < 0.05$ and $p < 0.01$) between mean values. n.s. not significant

Supplementary Figure 4

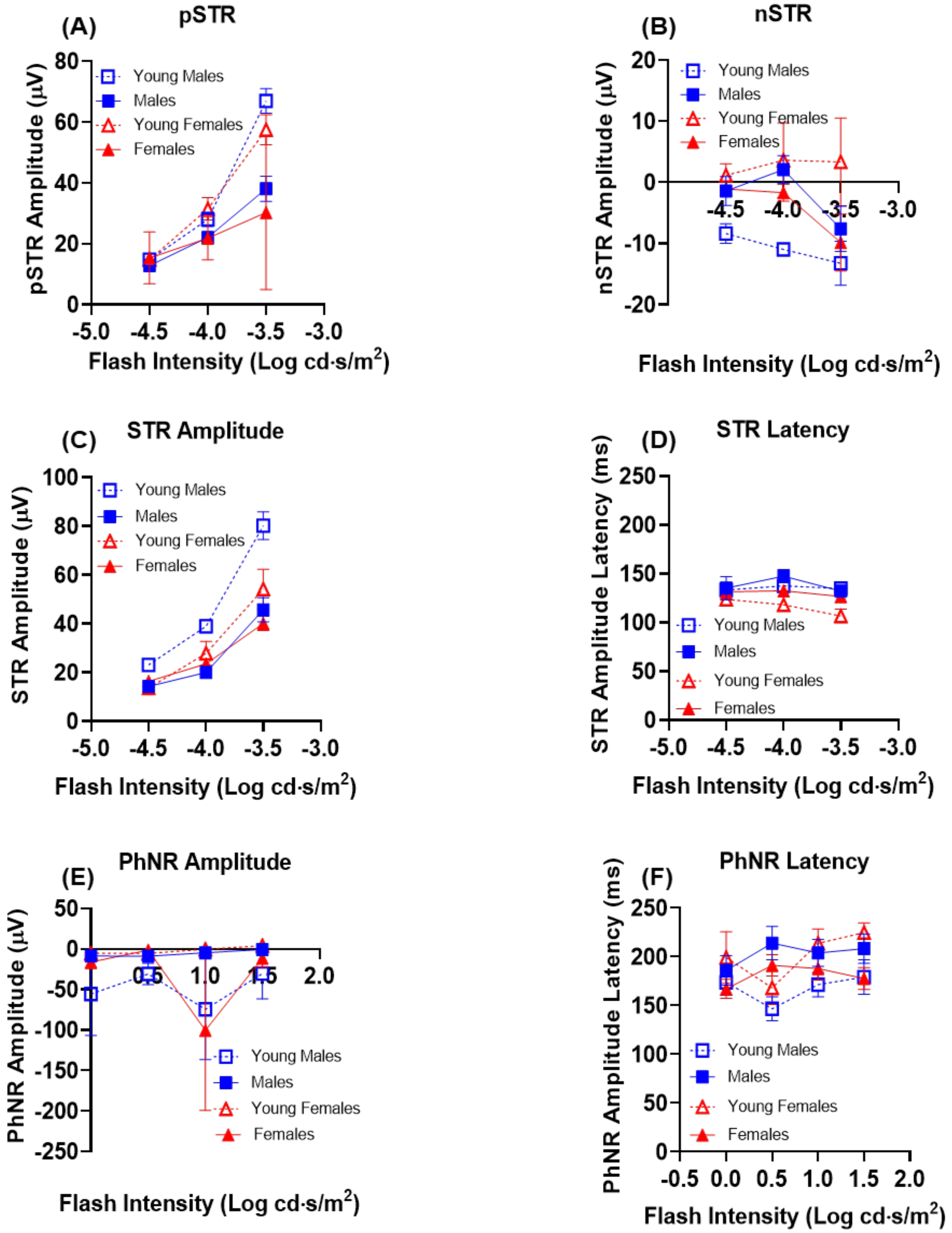

Supplementary Fig. 4: Age and sex-specific differences in threshold responses for scotopic and photopic ERG recordings. The pSTR (A), nSTR (B), STR amplitude (C), STR latency (D), PhNR amplitude (E), and PhNR latency (F) were calculated from responses in 15-month-old C57Bl/6J age-matched male and female mice eyes. Blue squares with solid blue lines symbolize individual male mice eyes (n=19), and red triangles with solid red lines symbolize individual female mice eyes (n=29). Individual male mice eyes (n=10) and individual female mice eyes (n=8) of 5-month-old C57Bl/6J mice were used as comparisons (open blue squares with dashed lines and open red triangles with dashed lines). Data are presented as mean ± SEM. Two-way ANOVA with Tukey's post-hoc test was used to determine the statistical significance of mean values for each group of mice and recorded in Supplementary Table 1. Comparisons not included in the table are not significant.

Supplementary Table 1. STR and PhNR ERG Components of Aging vs Young C57Bl/6J Mice

**pSTR**			
**-3.5**	**Males vs. Young Males**	********	**<0.0001**
**-3.5**	**Females vs. Young Females**	********	**<0.0001**

**STR Amplitude**			
**-4.0**	**Males vs. Young Males**	******	**<0.01**
**-3.5**	**Males vs. Young Males**	********	**<0.0001**
**-3.5**	**Females vs. Young Females**	******	**<0.01**
**-3.5**	**Young Males vs. Young Females**	*******	**<0.001**

**PhNR Latency**			
**0.5**	**Males vs. Young Males**	*****	**<0.05**

Flash intensities are expressed as log cd·s/m^2. Abbreviations: pSTR, positive scotopic threshold response; STR, scotopic threshold response; PhNR, photopic negative response

Supplementary Figure 5

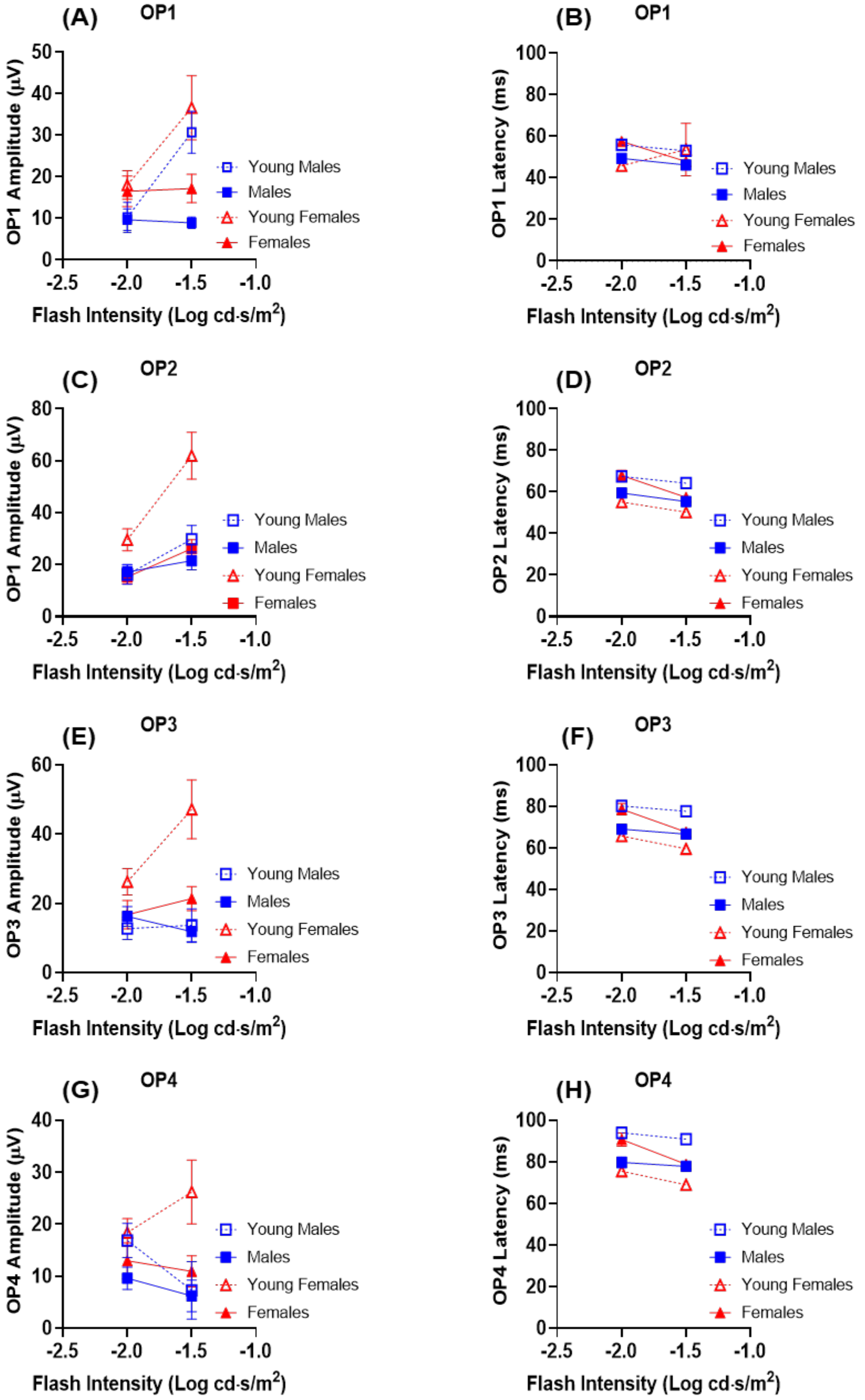

Supplementary Fig. 5: Age- and sex-specific oscillatory potential differences in ERGs from middle-aged C57Bl/6J mice. Scotopic oscillatory potentials were measured for amplitude and latency at four peaks labeled OP1 (A-B), OP2 (C-D), OP3 (E-F), and OP4 (G-H). Blue squares with solid blue lines symbolize individual male mice eyes (n=19), and red triangles with solid red lines symbolize individual female mice eyes (n=29). Individual male eyes (n=10) and individual female eyes (n=8) of 5-month-old C57Bl/6J mice were used as comparisons (open blue squares with blue dashed lines and open red triangles with red dashed lines). Data are presented as mean ± SEM. Two-way ANOVA with Tukey's post-hoc test was used to determine the statistical significance of mean values for each group of mice and recorded in Supplementary Table 2. Comparisons not included in the table are not significant.

Supplementary Table 2. Oscillatory Potentials of Aging vs. Young C57Bl/6J Mice

**Oscillatory Potential 1 Amplitude**			
**-1.5**	**Males vs. Young Males**	**	**<0.01**
**-1.5**	**Females vs. Young Females**	**	**<0.01**

**Oscillatory Potential 2 Amplitude**			
**-1.5**	**Females vs. Young Females**	****	**<0.0001**
**-1.5**	**Young Males vs. Young Females**	***	**<0.001**

**Oscillatory Potential 2 Latency**			
**-2.0**	**Males vs. Females**	*	**<0.05**
**-2.0**	**Females vs. Young Females**	**	**<0.01**
**-2.0**	**Young Males vs. Young Females**	**	**<0.01**
**-1.5**	**Males vs. Young Males**	*	**<0.05**
**-1.5**	**Young Males vs. Young Females**	***	**<0.001**

**Oscillatory Potential 3 Amplitude**			
**-1.5**	**Females vs. Young Females**	*	**<0.05**
**-1.5**	**Young Males vs. Young Females**	****	**<0.0001**

**Oscillatory Potential 3 Latency**			
**-2.0**	**Males vs. Females**	*	**<0.05**
**-2.0**	**Males vs. Young Males**	*	**<0.05**
**-1.5**	**Males vs. Young Males**	*	**<0.05**

**-1.5**	**Young Males vs. Young Females**	********	**<0.0001**

**Oscillatory Potential 4 Amplitude**			
**-1.5**	**Females vs. Young Females**	*****	**<0.05**
**-1.5**	**Young Males vs. Young Females**	*****	**<0.05**

**Oscillatory Potential 4 Latency**			
**-2.0**	**Males vs. Females**	*****	**<0.05**
**-2.0**	**Males vs. Young Males**	******	**<0.01**
**-2.0**	**Females vs. Young Females**	******	**<0.01**
**-2.0**	**Young Males vs. Young Females**	*******	**<0.001**
**-1.5**	**Males vs. Young Males**	******	**<0.01**
**-1.5**	**Young Males vs. Young Females**	********	**<0.0001**

Flash intensities are expressed as log cd·s/m^2. Abbreviations: OP, oscillatory potential

Supplementary Figure 6

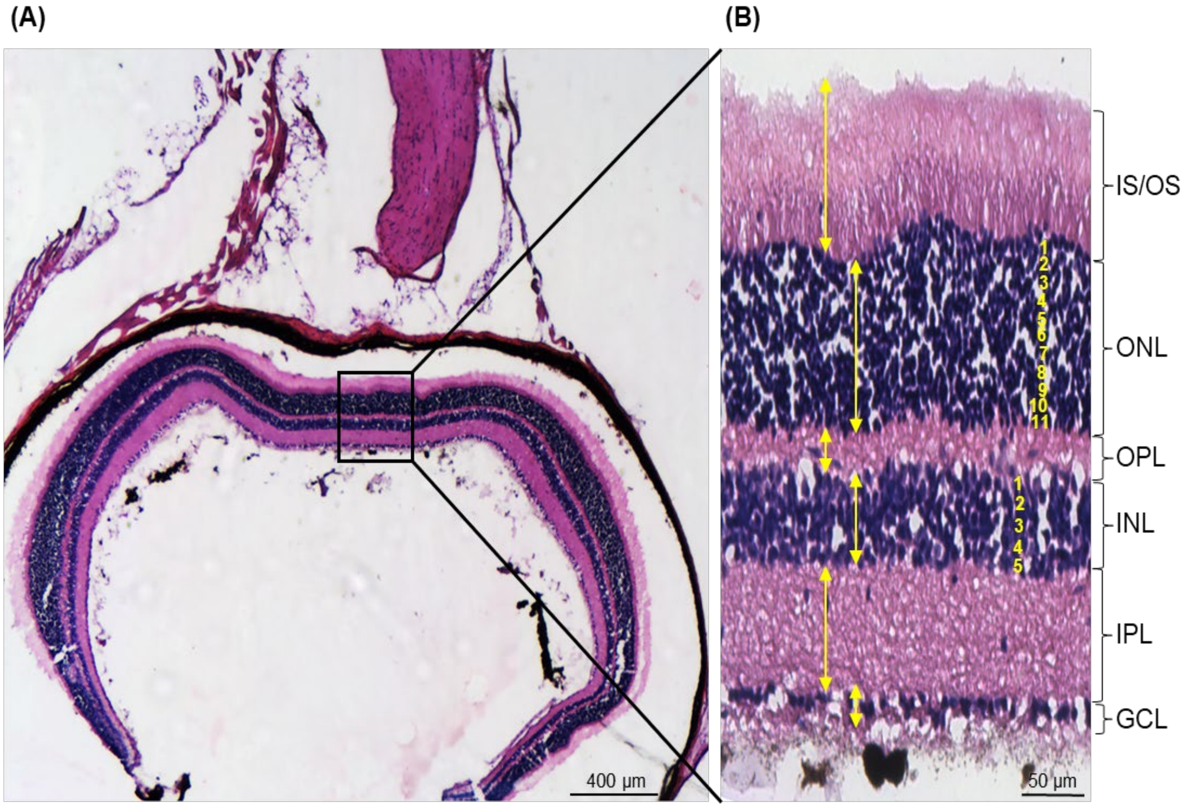

Supplementary Fig. 6: Depiction of the method used to quantify retinal thickness and number of nuclear rows. A 15-month-old C57Bl/6J female retina with severe vision loss (A). Demarcation of retinal layers is indicated by yellow bidirectional arrows, demonstrating how the boundaries are determined for thickness measurement of ganglion cell layer (GCL), inner plexiform layer (IPL), inner nuclear layer (INL), outer plexiform layer (OPL), outer nuclear layer (ONL), and inner segment/outer segment (IS/OS) layer. The section of retina outlined in black (A) is magnified and shown in (B), demonstrating how rows of nuclei are counted from the ONL and INL and numbered consecutively in yellow for each layer. H & E, 4x magnification, and scale bar = 400 µm for the eye cup panel, and 20x magnification and scale bar = 50 µm for the retinal layer panel.
